# The RNA-binding protein Igf2bp3 is critical for embryonic and germline development in zebrafish

**DOI:** 10.1371/journal.pgen.1009667

**Published:** 2021-07-02

**Authors:** Yin Ho Vong, Lavanya Sivashanmugam, Rebecca Leech, Andreas Zaucker, Alex Jones, Karuna Sampath

**Affiliations:** 1 Division of Biomedical Sciences, Warwick Medical School, University of Warwick, Coventry, United Kingdom; 2 School of Life Sciences, University of Warwick, Coventry, United Kingdom; 3 Centre for Early Life, University of Warwick, Coventry, United Kingdom; 4 Centre for Mechanochemical Cell Biology, University of Warwick, Coventry, United Kingdom; University of Pennsylvania School of Medicine, UNITED STATES

## Abstract

The ability to reproduce is essential in all branches of life. In metazoans, this process is initiated by formation of the germline, a group of cells that are destined to form the future gonads, the tissue that will produce the gametes. The molecular mechanisms underlying germline formation differs between species. In zebrafish, development of the germline is dependent on the specification, migration and proliferation of progenitors called the primordial germ cells (PGCs). PGC specification is dependent on a maternally provided cytoplasmic complex of ribonucleoproteins (RNPs), the germplasm. Here, we show that the conserved RNA-binding protein (RBP), Igf2bp3, has an essential role during early embryonic development and germline development. Loss of Igf2bp3 leads to an expanded yolk syncytial layer (YSL) in early embryos, reduced germline RNA expression, and mis-regulated germline development. We show that loss of maternal Igf2bp3 function results in translational de-regulation of a Nodal reporter during the mid-blastula transition. Furthermore, maternal *igf2bp3* mutants exhibit reduced expression of germplasm transcripts, defects in chemokine guidance, abnormal PGC behavior and germ cell death. Consistently, adult *igf2bp3* mutants show a strong male bias. Our findings suggest that Igf2bp3 is essential for normal embryonic and germline development, and acts as a key regulator of sexual development.

## Author summary

Animals develop from a single egg cell that is fertilised by sperm, to form various tissues and organs. How the different tissues form is still not fully understood. In particular, we don’t know precisely how organisms develop to be males or females, or how balanced sex ratios are maintained in populations. However, if these processes are disrupted, either too many males or too many females could arise in populations, which can lead to failure to produce the next generation, eventually resulting in extinction of the species. Therefore, identifying the factors that allow these processes to happen properly and deciphering how they function is crucial. We have identified a protein called Igf2bp3 in the tropical fish, Zebrafish, which if disabled, leads to most of the zebrafish developing as males. In addition, the few females that do make it to adulthood produce eggs that are defective, leading to abnormal progeny that generally do not survive. Our work identifies an important factor in fish that determines normal development and balanced sex ratios. Igf2bp3 has counterparts in other animals and therefore, our work can shed light on how these processes are controlled across organisms.

## Introduction

In eukaryotes, nascent RNA transcripts are bound in complexes of RNA-binding proteins (RBPs) known as heterogeneous nuclear ribonucleoproteins (hnRNPs) for further processing [1]. The purpose of these complexes are to direct the fate of its cargo RNAs, such as splicing [2,3], capping activity [4], polyadenylation [5] and export [6]. The composition of the hnRNPs contributes to the fate of cargo molecules. For example, hnRNPs containing the RNA-binding protein Igf2bp1 impart increased stability of bound RNAs, such as *c-myc* [7–9]. Mis-regulation of RNAs through abnormal RBP functions often lead to developmental consequences. For instance, knockout of mouse *Igf2bp1* leads to gross defects in development [10] and knockout of the RBP Zar1 in zebrafish leads to abnormalities in sex determination, resulting in male-only fish [11]. Several questions remain regarding how individual RNA-binding proteins contribute to development and the mechanism for their actions in specific cell types.

The germline is one of the earliest cell lineages to be specified across many multicellular organisms [12,13]. The precursor of the germline is the primordial germ cell (PGC). In mammals, the germline forms in the developing embryo, a process which is induced by signals from neighbouring cells [14]. In other animals, including fruit flies and zebrafish, a substance termed ‘germplasm’ is inherited by the egg from the mother, and contains factors that promote the formation of germline cells and development of the gonads.

Primordial germ cells receiving germplasm maintain their fate independently of the surrounding soma, whilst proliferating and migrating to the gonadal ridges [15]. Therefore, germplasm components are tightly regulated, and key components, including ribonucleoprotein complexes, are thought to be essential for correct PGC behaviour [16–18]. Germplasm RNAs are post-transcriptionally regulated by factors including microRNAs and RNA-binding proteins. RNA-binding proteins have a wide range of roles in processes such as oocyte maturation [11,19] and axis formation [20,21].

In addition to the functions that RNA-binding proteins have when bound to their target RNAs, the mechanism of the interactions is also important. For example, Ybx1 protein acts as a translational repressor and localisation factor for RNAs during early zebrafish embryogenesis. Ybx1 interacts with a stem-loop element found in the 3’ UTR of its target transcripts such as *sqt/ndr1* [21–24]. The DAZ family of proteins, which are critical for germline formation [25], interact with target RNAs via a specific sequence motif, and acts as translational activators [26,27]. More recently, post-transcriptional modifications to RNA are proposed to act as another marker for recognition by RNA-binding proteins. The modifications include N6-methyladenosine (m6A), which is recognised by a host of proteins such as Ythdc2 and Ythdf2, which have been proposed to have a role in germline development [28–31].

Another group of proteins that are now known to recognise m6A modifications is the Insulin-growth factor 2 mRNA binding protein (Igf2bp) family [32]. Igf2bp family members are nucleocytoplasmic proteins containing two RNA-recognition motifs (RRMs) and two K-Homology (KH) didomains [33] that are highly conserved. Igf2bp proteins have many diverse functions. In addition to acting as RNA stabilisers [32,34], Igf2bp proteins also have roles in localisation. For instance, *Xenopus* Igf2bp3 binds to a *vg1* 3’ UTR element to localise *vg1* transcripts to the vegetal cortex of oocytes [35–37]. Subsequent work has shown Igf2bp proteins to play a role in development in *Xenopus* [38], mice [10] and zebrafish [34]. However, whether Igf2bp proteins have a role in vertebrate germline development remains unexplored, although many lines of evidence point towards this possibility. In zebrafish oocytes, zebrafish *igf2bp3* transcript is enriched in the germplasm-containing structure, the Balbiani body [20], and *Drosophila igf2bp* has been shown to regulate germ cell maintenance [39].

To determine the role of Igf2bp3 in zebrafish development, we generated and analysed zebrafish mutants affecting *igf2bp3*, and find that maternal Igf2bp3 is essential for proper germline development. Loss of zygotic *igf2bp3* is sufficient to induce a male-sex bias in adult zebrafish. Loss of Igf2bp3 leads to a reduction in canonical germline markers, and maternal *igf2bp3* mutants have a mis-regulated germline, with depletion of primordial germ cells and aberrant PGC behaviour. These results highlight a new function for this family of proteins in germline development and identify a key maternal factor that is essential for germline development, maintenance and PGC migration.

## Methods

### Ethics statement

Adult zebrafish were kept at their ambient temperature [40] in the University of Warwick aquatics facility, in compliance with the University of Warwick animal welfare and ethical review board (AWERB) and the UK home office animal welfare regulations, covered by the UK Home office licenses PPL 70/7836 and P782A73C4 to KS, and PEL 30/2308 and X59628BFC to the University of Warwick.

### Animal husbandry

Embryos were collected from pair-wise or pooled mating of adult wild type or mutant zebrafish and incubated at 28.5°C in 0.3X Danieau’s solution with methylene blue (17 mM NaCl, 2 mM KCl, 0.12 mM MgSO_4_, 1.8 mM, Ca(NO_3_)_2_, 1.5 mM HEPES, pH 7.6). Embryos were manually dechorionated with a pair of Dumont #5 tweezers.

### Affinity purification, mass spectrometric analysis and identification of Igf2bp3

NHS (N-hydroxysuccinimide) activated Sepharose 4 fastflow (GE Healthcare) beads coupled with an RNA aptamer alone control or aptamer fusion (zebrafish *ndr1* 3’UTR sequences fused to a Tobramycin aptamer; S1 Methods) was incubated with 10 mg of 20 min post-fertilisation embryonic lysates, washed and eluted with Tobramycin antibiotic [41,42]. The eluates were run on 5–16% gradient polyacrylamide gel, washed with deionized water and stained with Coomassie blue for 15 minutes. Protein bands were cut into cubes of ∼1mm. Gel pieces were de-stained twice using 50% ethanol (Thermo Fisher Scientific) in 50 mM ammonium bicarbonate (ABC, Fluka) at 22°C for 15 min and dehydrated with 100% ethanol for 5 min with shaking (650 rpm). Dehydrated gel pieces were reduced with 10 mM DTT (Sigma) at 55°C for 30 min and then alkylated with 55 mM iodoacetamide (Sigma) for 20 min in the dark at 22°C. Samples were then washed with 50% ethanol in 50 mM ABC at 22°C for 15 min and then dehydrated with 100% ethanol for 5 min. The gel pieces were hydrated and incubated with 2.5 ng/μl of trypsin (Promega) in 50 mM ammonium bicarbonate (ABC) overnight at 37°C. Peptides were extracted from gel pieces three times with 5% formic acid in 25% acetonitrile (ACN; Thermo Fisher Scientific) with 5-minute sonications in a water bath. The supernatants were combined in a fresh vial, dried using a vacuum centrifuge at 40°C, resuspended in 55 μl of 2.5% acetonitrile containing 0.05% trifluoroacetic acid and sonicated for 30 mins. 20μl of the sample was used for liquid chromatography-mass spectrometry analysis as performed previously [43]. Samples were analysed using reversed phase chromatography with two columns, an Acclaim PepMap μ-precolumn cartridge 300 μm i.d. x 5 mm, 5 μm, 100 Å and an Acclaim PepMap RSLC 75 μm i.d. x 50 cm, 2 μm, 100 Å (Thermo Scientific) was used to separate tryptic peptides prior to mass spectrometric analysis. The columns were installed on an Ultimate 3000 RSLCnano system (Dionex) at 40°C. Mobile phase buffer A composed of 0.1% formic acid and mobile phase B composed of acetonitrile containing 0.1% formic acid. Samples were loaded onto the μ-precolumn equilibrated in 2% aqueous acetonitrile containing 0.1% trifluoroacetic acid for 5 min at 10 μL min-1, after which peptides were eluted onto the analytical column at 250 nL min-1 by increasing the mobile phase B concentration from 6% B to 37% over 100 min, followed by a 3 minute wash at 80% B and a 10 min re-equilibration at 4% B. Eluting peptides were converted to gas-phase ions by means of electrospray ionization and analysed on a Thermo Orbitrap Fusion (Thermo Scientific). Survey scans of peptide precursors from 375 to 1500 m/z were performed at 120K resolution (at 200 m/z) with a 2x105 ion count target. The maximum injection time was set to 150 ms. Tandem MS was performed by isolation at 1.2 Th using the quadrupole, HCD fragmentation with normalized collision energy of 33, and rapid scan MS analysis in the ion trap. The MS2 ion count target was set to 5x103. The maximum injection time was 200 ms. Precursors with charge state 2–6 were selected and sampled for MS2. The dynamic exclusion duration was set to 40 s with a 10 ppm tolerance around the selected precursor and its isotopes. Monoisotopic precursor selection was turned on. The instrument was run in top speed mode. The acquired tandem mass spectra, as Xcalibur (version 2.2) raw files were analysed using Max-Quant software v1.6.0.16 [44,45] against UniProtK *Danio rerio* (UP000000437). Trypsin was specified as the digestion enzyme, with up to 2 missed cleavages, and a parent ion mass tolerance of 4.5 ppm for the initial search, with recalibration enabled. Oxidation of methionine was set as a variable modification and carbamidomethyl of cysteine as a fixed modification for all searches. The MS-MS data were further collated using Scaffold software. Fold change in proteins between the control aptamer and zebrafish ndr1 fusion RNA samples were calculated using emPAI in scaffold. The emPAI is a label-free, relative quantitation of the proteins in a mixture based on protein coverage by the peptide matches in a database search result. Igf2bp3 showed > 4-fold enrichment in ndr1-bound eluates.

### Generation and establishment of *igf2bp3* mutants

Cas9 mutants were generated in the TU background. Target sequences were verified by PCR sequencing and the conserved *igf2bp3* cDNA sequence was used as a target in CHOPCHOP [46] to produce the target site “GGCTCCCTTCCTCGTAAAAAG” in exon 1. Embryos were injected with capped 150 pg Cas9 RNA and 50 pg sgRNA at the 1-cell stage, and raised to adulthood as described [47]. F0s were outcrossed to identify heterozygous F1s, which were outcrossed again to expand the line, followed by mating of heterozygous sibling to retrieve homozygous mutants. The *igf2bp3* transgenic insertion alleles were obtained from the Lin/Burgess retroviral insertion collection, outcrossed over two generations and subsequently, heterozygous carriers were inter-crossed to homozygosity. Experiments were typically carried out with at least two females, and results from one representative clutch are shown.

### Genotyping mutants

DNA was isolated from fin clips by incubating with lysis buffer (10 mM Tris pH 8.3, 50 mM KCl) with proteinase K (200 μg/mL) at 55°C overnight, followed by inactivation at 95°C for 10 minutes and lysates were used directly for PCR (primer sequences provided in S1 Methods). PCR products were visualised on a 2–3% agarose gel. The *igf2bp3*^*la020659Tg*^ insertion mutant fish were genotyped by the use of three primers in a single PCR reaction, a forward/reverse primer flanking the insertion and a second reverse primer specific to the long terminal repeats in the retroviral construct. This generates a 250 bp WT product and a ~800 bp product for the insertion allele. The *igf2bp3*^*la010361Tg*^ insertion allele was genotyped by the use of three primers in a single PCR reaction, a forward/reverse primer flanking the insertion and a second forward primer specific to the long terminal repeats in the retroviral construct. This generates a 350 bp WT product and a ~900 bp product for the insertion allele. The *igf2bp3*^*Δ7*^ deletion allele contains a continuous 7 bp deletion in exon 1. This mutation generates a BssaI (NEB) restriction enzyme site.

### Synthesis of RNAs

Synthetic capped mRNAs were transcribed using the SP6 mMessage mMachine, following the manufacturer’s instructions. Cas9 sgRNA was transcribed using the T7 HiScribe High Yield RNA Synthesis kit, following the manufacturer’s instructions. RNAs were purified with phenol-chloroform extraction. The pSP64-mmGFP5-nos1-3’UTR [48] and pSP64-eGFP-F-nos1-3’UTR [49] constructs were linearised with SacII and NotI restriction enzymes (NEB) respectively, following the manufacturer’s instructions. These were used to label the cytoplasm and cell membranes of the PGCs. Probes for whole mount *in situ* hybridisation were produced with Promega SP6/T7/T3 polymerases with DIG-labelling mix (Roche) and purified with lithium chloride extraction.

### Whole mount *in situ* hybridisation

Embryos were fixed in 4% paraformaldehyde in PBS (phosphate buffered saline), and processed for whole mount *in situ* hybridization (WISH) to detect gene expression as described previously [50]. Experiments were typically carried out with at least two females and we analysed independent clutches from each female. Results from one female are shown for *gdf3* and *wnt8a*, whereas the *dazl* WISH represents two clutches from two independent females, which showed 12/35 (34%) and 15/29 (52%), respectively.

### Total RNA extraction and qRT-PCR

Embryos were collected at various stages and lysed in TRIzol, followed by RNA extraction using the Monarch Total RNA Miniprep kit according to the manufacturer’s instructions. For the one cell stage, maternal, maternal/zygotic *igf2bp3* mutants in *Tg*(*buc*:*buc-egfp*); *buc106*+/- background or maternal/zygotic *igf2bp3* mutants were used. For 1K and 50% epiboly stages, maternal/zygotic *igf2bp3* mutants in *Tg*(*buc*:*buc-egfp*); *buc106*+/- background or maternal/zygotic *igf2bp3* mutants alone were used. Embryos were collected and incubated at 28.5°C until the appropriate stage for RNA extraction. First strand cDNA was synthesised using the qPCRBIO cDNA Synthesis kit and qPCR performed (primer sequences provided in S1 Methods) using the qPCRBIO SyGreen Blue Mix Lo-ROX or Luna Universal qPCR Master Mix. Analysis was performed using the Stratagene Mx3005P.

### Protein gel electrophoresis and Western blot

SDS-PAGE gels were prepared using the Bio-Rad protein electrophoresis systems. Zebrafish embryos were homogenised in RIPA (50 mM Tris-HCl, 150 mM NaCl, 1% (v/v) NP-40, 0.5% (w/v) sodium deoxycholate, 1 mM EDTA, 0.1% (w/v) SDS) lysis buffer supplemented with protease inhibitor cocktail (Sigma-Aldrich) using a syringe and needle. Supernatants were collected after a brief centrifugation and boiled in 4X loading buffer (200 mM Tris-HCl (pH 6.8), 400 mM DTT, 8% SDS, 0.4% bromophenol blue and 40% glycerol) before loading. After transfer of proteins, membranes were rinsed with TBSTw (Tris Buffered Saline, 0.1% Tween-20) once and blocked in 5% skimmed-milk powder in TBSTw for 1 hour before incubation with primary antibody overnight. After incubation, membranes were rinsed 4X in TBSTw for 5 minutes and transferred to secondary antibody for 4 hours at room temperature, excess antibody subsequently removed by a further 4 washes in TBSTw for 5 minutes before detection with ECL Western blotting reagent (Bio-Rad) following manufacturer’s instructions. Signal detection was performed using a ChemiDoc MP Imaging system (Bio-Rad) or with CL-XPosure Film (34089, Thermo Fisher Scientific).

### Image acquisition

For live tracking of PGCs, embryos were mounted in 0.8% LMPA on a heated stage maintained at 28.5°C and imaged with an Andor Revolution Spinning Disk system, based on a Nikon Ni-E PFS inverted microscope equipped with a Yokogawa CSU-X1 spinning disk unit, fitted with a 488 nm laser and captured with an iXon Ultra 888 EMCCD camera. Images were captured using either a Nikon Plan Apochromat 20X/0.75 NA or the Nikon Apochromat 60X/1.49 NA oil immersion objectives. Images were acquired with the Andor iQ3 software.

For time-lapse imaging of migrating PGCs, images were acquired using a 20X objective and z-stacks were generated with 1μm step-sizes and at 1-minute intervals for 60 minutes. Maximum intensity projections were generated using ImageJ/Fiji, and the MTrackJ plugin was used to derive displacement, speed and straightness of PGCs [51].

For analysing PGC filopodia dynamics, z-stacks were generated with the 60X objective with 0.5 μm step-sizes at 10 second intervals for 2–10 minutes. Using ImageJ/Fiji, maximum intensity projections were generated and the filopodia numbers per PGC were recorded by counting the filopodia for a single timepoint. Persistence of the filopodia was calculated by counting the number of consecutive frames that a single filopodium was present. The length of a filopodium was calculated as the average length over its observable lifetime. The frequency of filopodia projections was calculated by measuring the direction of the projections relative to the embryonic midline. To image PGCs *in vivo* during segmentation, the Zeiss LSM 880 scanning confocal microscope was used.

### Immunofluorescence and TUNEL assay

To detect the YSL, the cell membranes were labelled with an anti-β-Catenin antibody (C2206, Sigma Aldrich) and nuclei were labelled with DAPI stain as described [21]. The embryos were washed in PBS, mounted in 1% low melting agarose in PBS, and imaged with a Zeiss LSM 880 scanning confocal microscope, using 25X Zeiss Plan-Neofluar 25X/0.8 NA and 40X Plan-Neofluar 40X/1.3 NA objective lenses.

For TUNEL labelling of PGCs, embryos were fixed and processed for immunofluorescence using an anti-Vasa antibody (ab209710, Abcam) as described [52], and subsequently processed for TUNEL labelling following the manufacturer’s instructions (Sigma Aldrich).

### Nodal/Squint translation assay

MZ*igf2bp3*^*la659Tg*^ mutant and control embryos were obtained from crosses of adult mutants and either heterozygous carriers or wild type siblings, respectively. For detection of Sqt-GFP reporter at high stage, embryos derived from crosses of heterozygous siblings were used as controls. TU WT embryos served as controls for detection of ΔΥΒΕ-Sqt-GFP at high stage. Synthetic mRNAs for microinjections were synthesized using the mMessage mMachine SP6 kit with Not I linearised pCS2+ plasmids containing either *sqt-gfp:sqt*3’UTR or a *sqt-gfp*:ΔYBE 3’UTR control construct lacking the YBE sequence. The fusion constructs harbour a 3xFLAG:enterokinase cleavage site cassette [53,54] fused to EGFP one amino acid after the pro-protein convertase cleavage site RRHRR, so that EGFP is fused to mature Nodal peptide after processing. Embryos were microinjected at the 1-cell stage with 100–150 pg of either *sqt-GFP* or *ΔYBE sqt-GFP* RNA [21,24]. Rhodamine dextran (0.5%, 70 kDa) was co-injected as an injection control for all experiments except high stage sqt-gfp injections. Injected embryos were dechorionated by Pronase treatment (2 mg/ml for 2 minutes) and incubated at 28.5°C until mounting and imaging. Embryos were mounted for lateral views in agarose-coated glass-bottom dishes and imaged on an Andor Revolution XD microscope equipped with an Andor iXon 888 camera. Z-stacks of 50 μm in 0.56–0.59 μm slices were acquired using a 488 nm laser at 40% and a 561 nm laser at 10%. Intensity measurements were carried out using Fiji software. Maximum intensity projections of the Z-stacks were generated. Threshold-segmentation in the rhodamine channel followed by “Analyse Particles” produced a single ROI corresponding to the area of the blastoderm that has been imaged. Mean pixel values in the ROI were measured in both channels. To normalize GFP signal, the mean GFP intensity was divided by the mean rhodamine intensity. Bar plots were produced in R-studio.

### Injections with *igf2bp3* mRNA

To rescue the MZ*igf2bp3*^*la659Tg*^ and MZ*igf2bp3*^*Δ7*^ phenotypes, we injected *igf2bp3* mRNA made from a pCS2+ *igf2bp3* cDNA plasmid that was linearised with NotI. Capped mRNA was generated using the mMessage mMachine SP6 kit, and 1-cell stage mutant or control embryos were injected with 100 or 200 pg *igf2bp3* mRNA. Similarly, *igf2bp1* RNA was generated and injected. Injected embryos were scored for YSL expansion at the 1K cell stage, or fixed at prim5 for WISH to detect *ddx4/vasa* expression.

### Statistical analysis

Data analysis was typically performed with two-tailed t-tests, except for analysis of sex ratios in *igf2bp3* mutants, which was performed with a Binomial test [55].

## Results

### Mutations in *igf2bp3* result in biased sex ratios in adult zebrafish

Igf2bp3 was identified from a proteomic screen to identify RNA-binding proteins in early zebrafish embryonic lysates that bind an RNA aptamer. Igf2bp3 was enriched 4-fold compared to controls. Analysis of *igf2bp3* expression by WISH and available RNAseq datasets shows that *igf2bp3* is ubiquitously expressed and at high levels during early development (S1A and S1B Fig). Igf2bp3 protein is expressed maternally and zygotically (S2C Fig), precluding the use of morpholinos to uncover the role of *igf2bp3* in development.

**Fig 1:**
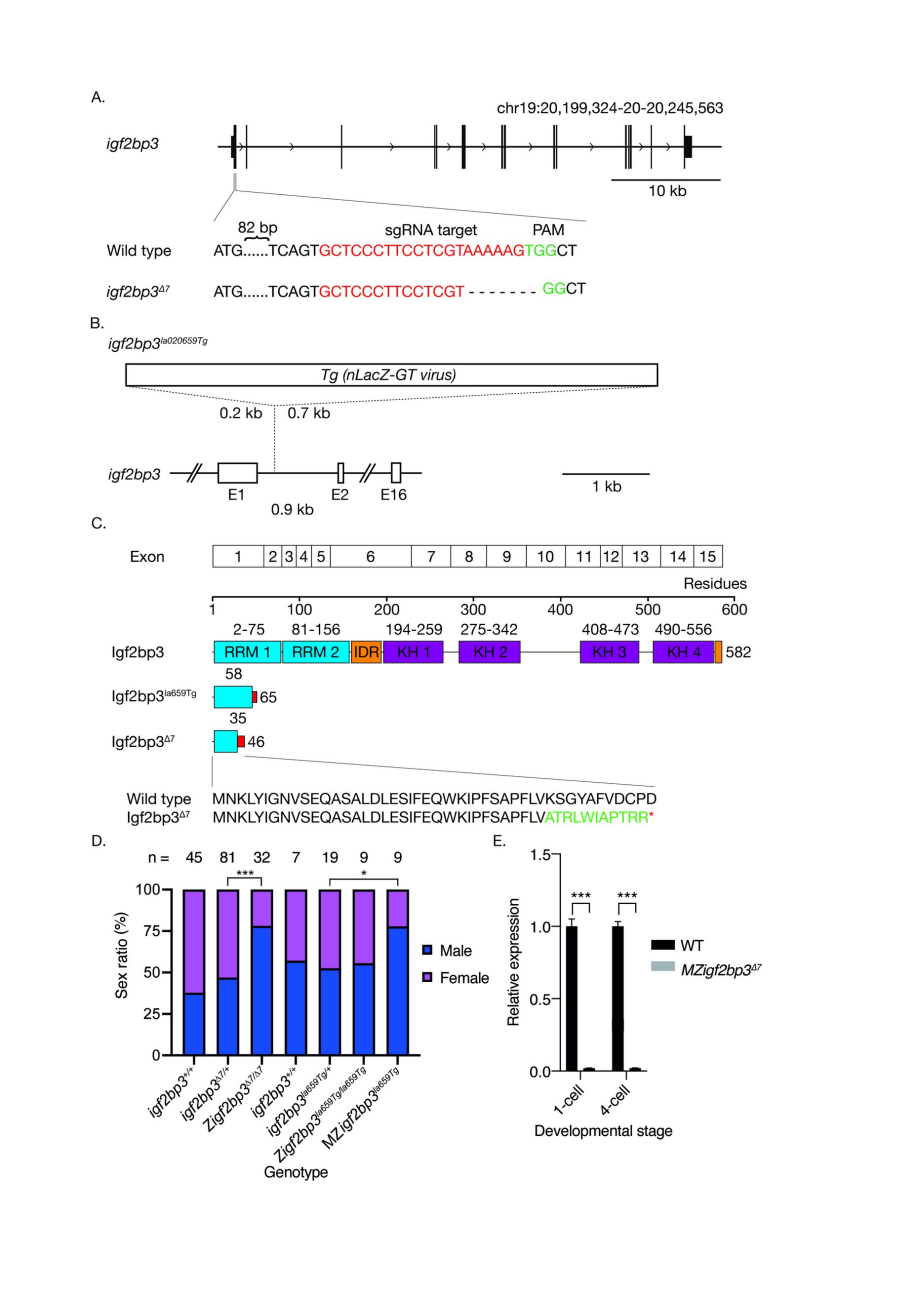
Loss of *igf2bp3* results in skewed male:female sex ratios. A. Generation of the *igf2bp3* mutant allele by Cas9. Schematic showing the *igf2bp3* locus on chromosome 19. Exon 1 of *igf2bp3* was targeted by CRISPR-Cas9, resulting in a 7 bp deletion and frameshift. B. Schematic of the *igf2bp3*^*la659Tg*^ with a 6 kb Tg(*nLacZ-GTvirus*) retroviral insertion in intron 1. C. The *igf2bp3*^*Δ7*^ mutant is predicted to be protein null. The *igf2bp3*^*Δ7*^ mutation generates a premature stop codon at residue 46 whereas the *igf2bp3*^*la659Tg*^ results in a predicted truncated protein of 65 amino acids. D. Loss of *igf2bp3* results in skewed sex ratios. Adult zygotic *igf2bp3*^*Δ7*^ and MZ*igf2bp3*^*la659Tg*^ mutant fish show a significant male bias compared to wild type, heterozygous *igf2bp3*^*Δ7*^ as well as *igf2bp3*^*la659Tg*^, and Z*igf2bp3*^*la659Tg*^ fish. Asterisks indicate level of significance (p *<0.05, **<0.01, ***<0.001). E. The *igf2bp3*^*Δ7*^ mutation results in reduced *igf2bp3* levels. qRT-PCRs at the 1-cell and 4-cell stages show significantly reduced *igf2bp3* transcript levels in *igf2bp3*^*Δ7*^ mutant embryos (p<0.001).

To identify zebrafish lines with genomic lesions in *igf2bp3*, we searched a mutant collection generated by the Lin and Burgess laboratories [56]. We identified several retroviral transgenic insertion alleles that harbour integration of the *Tg(nLacZ-GTvirus)* in intron one of the *igf2bp3* locus: *igf2bp3*^*la010361Tg*^ and *igf2bp3*^*la020659Tg*^, hereafter referred to as *igf2bp3*^*la361Tg*^ and *igf2bp3*^*la659Tg*^ (Fig 1B). The *igf2bp3* insertion mutant alleles are predicted to result in loss-of-function by preventing splicing of exons one and two in *igf2bp3* transcripts, leading to premature termination codons. RT-PCRs of exon-spanning junctions of *igf2bp3* showed a reduction of RNA levels (S2B Fig). Western blots performed using an antibody directed against a central region of Igf2bp3 downstream of the insertion site did not reveal any detectable Igf2bp3 in mutant embryo lysates (S2C Fig).

To generate *igf2bp3* null mutants, we used CRISPR-Cas9 mutagenesis, and identified mutants harbouring a 7 bp deletion within *igf2bp3* exon one, hereafter referred to as *igf2bp3*^*Δ7*^ (Fig 1A). The deletion is predicted to result in a frameshift at residue 35 that leads to a truncated Igf2bp3 peptide of 47 residues which lacks the RNA-binding domains and the predicted intrinsic disordered regions (Fig 1C), and results in a significant reduction in *igf2bp3* RNA (Fig 1E).

Zygotic *igf2bp3*^*la659Tg*^ embryos appear morphologically normal and grow to adults at expected Mendelian ratios (Figs 1D and S2E and S2F). However, progeny of *igf2bp3*^*la659Tg*^ adults (i.e., MZ*igf2bp3*^*la659Tg*^) show biased sex ratios as adults (Fig 1D), with an approximately 3:1 male-female ratio. We also observed a similar male sex bias (3:1) in zygotic *igf2bp3*^*Δ7*^ deletion mutant adults (Figs 1D and S1C), suggesting that Igf2bp3 might have important roles in normal germline, gonad and/or sexual development.

### Maternal Igf2bp3 plays a critical role in early development

**Fig 2:**
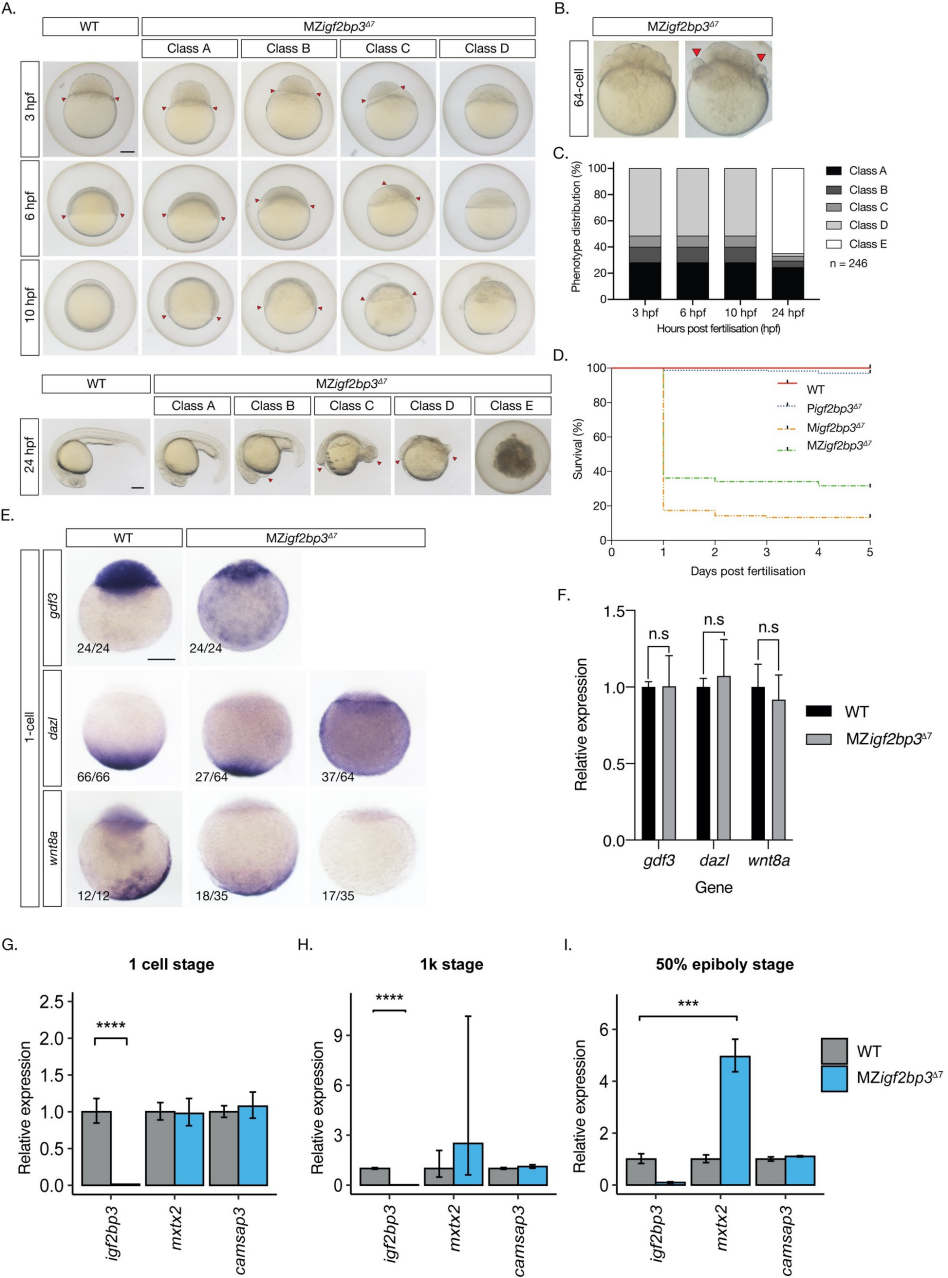
Maternal *igf2bp3* is required for normal embryonic development. A. Maternal *igf2bp3*^*Δ7*^ mutants show severe defects by blastula and gastrula stages. B. At the 64-cell stage, most maternal *igf2bp3*^*Δ7*^ mutants are similar to wild type embryos (left), but a proportion of embryos (right; <15%) show rounded cells (red arrowheads). C-D. The majority of *igf2bp3* mutants do not survive beyond 1 dpf. E. Animal-vegetal markers (*gdf3*, *dazl* and *wnt8a*) show altered distribution in *igf2bp3*^*Δ7*^ embryos by whole mount in situ hybridization (WISH). F. qRT-PCRs show that expression levels of *gdf3*, *dazl* and *wnt8a* transcripts is not significantly different from wild type control embryos at the 1-cell stage. G-I. qRT-PCR with maternal *igf2bp3*^*Δ7*^ embryos show that expression of *igf2bp3* transcripts is significantly reduced at all stages examined (1 cell, 1K, and 50% epiboly), whereas expression of the yolk syncytial layer (YSL) transcript *mxtx2* is initially unchanged (G), but shows variable increase at the 1K stage (F), with substantially higher expression at 50% epiboly compared to controls (I), and the microtubule and spectrin-associated *camsap3* transcript is largely unchanged at all stages examined. p ***<0.01 or ****<0.001 from 3 biological replicates. Scale bar in A, 200 μm.

Although zygotic *igf2bp3* mutants are viable up to adulthood, we observed defects in the progeny of homozygous *igf2bp3* mutant females, i.e., maternal *igf2bp3*^*Δ7*^ embryos. Early cell divisions and cleavage are similar to wild type embryos in the majority of maternal *igf2bp3* mutants (Fig 2B). However, a small proportion of deletion mutant embryos (10–15% from different females) show defects in cell adhesion at 32–64 cell stage, with some blastoderm cells rounding up and detaching from the embryo (red arrowheads, Fig 2B). These embryos later manifest severe phenotypes (described below). We observed gross defects across the blastoderm from the 1K-cell stage, resulting in delayed progression through gastrula and lethality in the majority of embryos by 24 hpf (Fig 2A–2D). We have categorised the embryos as class A (delayed progression of gastrulation), class B (abnormal blastoderm and expanded YSL), class C (pockets of blastomeres and massively expanded YSL), class D (severe). All class C and D embryos are lethal by 24 hpf (class E). The majority of these embryos are not viable and fail to progress beyond 24 hpf.

In maternal *igf2bp3*^*la659Tg*^ mutant embryos, early cell divisions are not affected (S2F Fig) and lethality is not observed, although mutant embryos are delayed briefly at late blastula stages. There is no visible morphological defect in MZ*igf2bp3*^*la659Tg*^ mutants, with expression of *myoD* in the myotome and *shh* in the midline comparable to wild type embryos and the swim bladder inflates normally in MZ*igf2bp3*^*la659Tg*^ embryos (S2D and S2E Fig). To determine if paralogue redundancy underlies the viability of the *igf2bp3*^*la659Tg*^ mutant embryos, we examined the expression of other *igf2bp* transcripts. Whereas *igf2bp3* expression is not detected in *igf2bp3*^*la659Tg*^ mutant embryos at all stages, we do not find an increase in the expression levels of *igf2bp1*, *igf2bp2a*, or *igf2bp2b* transcripts (S2G Fig). Therefore, paralogue gene expression is not altered in maternal *igf2bp3*^*la659Tg*^ mutant embryos.

### Distribution of maternal RNAs is disrupted in maternal *igf2bp3* mutant embryos

As Igf2bp3 is an RNA-binding protein that is involved in the regulation of *vg1* (now known as *gdf3*) in *Xenopus* and enriched in the Balbiani body, we examined maternal-zygotic *igf2bp3*^*Δ7*^ embryos at the 1-cell stage for expression of the animal pole marker *gdf3* and vegetal markers, *dazl* and *wnt8a*. MZ*igf2bp3*^*Δ7*^ embryos showed markedly reduced *gdf3* expression in the blastoderm, with some expression detected in the yolk of mutant embryos. Expression of *dazl* and *wnt8a* transcripts is either reduced or not detectable in the vegetal cortex, and a proportion of maternal mutant embryos show diffuse staining for *dazl* (Fig 2E). Quantitative real-time PCR (qRT-PCR) in 1-cell stage embryos show that the expression levels of *gdf3*, *dazl* and *wnt8a* in MZ*igf2bp3*^*Δ7*^ mutant embryos is not different from controls (Fig 2F). Therefore, it is likely that maternal Igf2bp3 plays a key role in the distribution, anchoring and/or stability of a subset of maternal RNAs.

### The extra-embryonic yolk syncytial layer (YSL) is expanded in maternal *igf2bp3* mutants

In maternal *igf2bp3*^*Δ7*^ as well as MZ*igf2bp3*^*la659Tg*^ mutant embryos at 3 hpf, we observed an expansion of the extra-embryonic yolk syncytial layer (YSL; class B,C in Figs 2A and S3A and S3B). Remarkably, this expansion is not observed in MZ*igf2bp3*^*la659Tg*^ at 4.5 hpf, and MZ*igf2bp3*^*la659Tg*^ mutant embryos recover to complete gastrulation and survive (S3A and S3B Fig). By contrast, the majority of maternal *igf2bp3*^*Δ7*^ embryos show an expanded YSL, a small blastoderm, defects during gastrulation and do not survive beyond 24 hpf (Fig 2A–2D). Consistent with these observations, quantitative real-time PCR (qRT-PCR) in early embryos show that the YSL transcript *mxtx2* is increased from the 1K-cell stage and is significantly increased at gastrula stages in MZ*igf2bp3*^*Δ7*^ embryos but in MZ*igf2bp3*^*la659Tg*^ *mxtx2* levels are either unchanged or slightly reduced (Figs 2G–2I and S3C–S3E). The microtubule and spectrin-associated *camsap3* transcript is unchanged in both mutants at all stages examined. Injection of *igf2bp3* mRNA into MZ*igf2bp3*^*Δ7*^ mutant embryos fails to rescue the YSL expansion (S4A Fig). Therefore, maternal Igf2bp3 plays an important role in the allocation of embryonic versus extra-embryonic cell fates and embryonic survival.

### Maternal Igf2bp3 mutant embryos show translational deregulation

**Fig 3:**
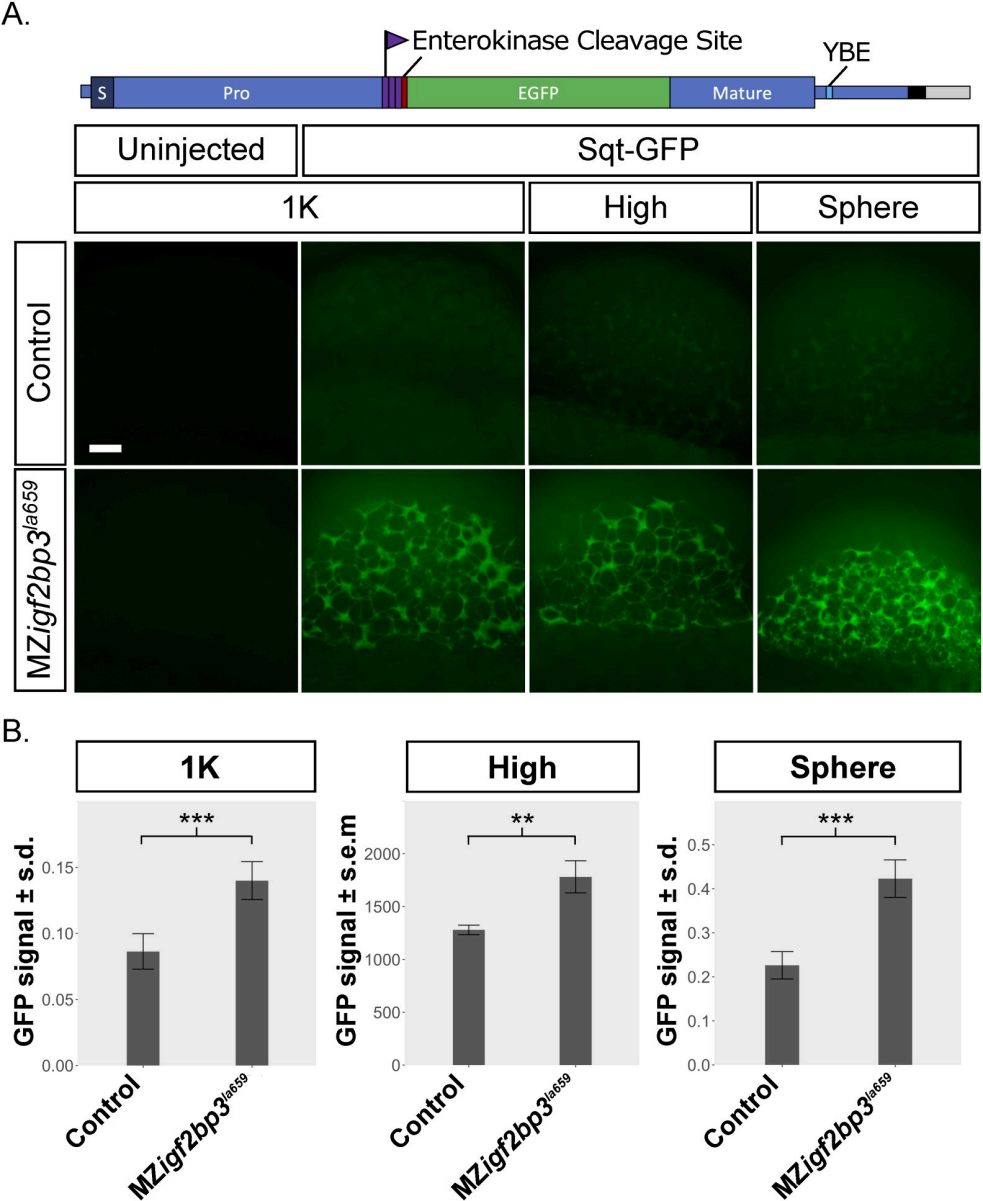
MZ*igf2bp3*^*la659Tg*^ mutant embryos show premature translation of a Nodal reporter. A. Schematic of the *sqt-gfp* fusion reporter construct showing the Sqt pro-domain, the enterokinase cleavage site, 3xFLAG epitope tag, eGFP and mature Nodal peptide sequences. Expression of a Sqt-GFP reporter expression (green) in MZ*igf2bp3*^*la659Tg*^ embryos compared to wild type or control heterozygous embryos. Representative examples from three independent experiments are shown, imaged at the 1K, high or sphere stage, respectively. Scale bar, 50 μm. B. Bar charts represent mean GFP intensity in the blastoderm of imaged embryos. GFP signals were normalized to co-injected rhodamine dextran control. Number of embryos analysed: 1K (n = 9 WT and 10 MZ*igf2bp3*^*la659Tg*^), high (n = 10 Control and 11 MZ*igf2bp3*^*la659Tg*^), sphere (n = 9 WT and 9 MZ*igf2bp3*^*la659Tg*^). Asterisks indicate level of significance from two-tailed t tests (p *<0.05, **<0.01, ***<0.001).

YSL expansion could potentially arise from cleavage or cytoplasmic segregation defects [57–59]. However, we did not see any defects in cell divisions or cleavage in mutant embryos for MZ*igf2bp3*^*la659Tg*^ or MZ*igf2bp3*^*Δ7*^ (Figs 2B and S2F). We identified Igf2bp3 as a component of an RNA-binding complex that recognizes the 3’ untranslated (3’UTR) region of *nodal/squint* RNA. Defects in another protein in the same RNP complex, Ybx1, also result in YSL expansion, which we have shown to arise from translational de-regulation and premature Nodal signaling in maternal *ybx1* mutant embryos [21]. We therefore investigated if maternal Igf2bp3 functions in translational control of *nodal/sqt* RNA. In MZ*igf2bp3*^*la659Tg*^ embryos injected with *sqt-gfp* fusion reporter RNA (Fig 3A), we found high levels of Sqt-GFP protein expression in the blastoderm at 1K, high and sphere stages. By contrast, control (wild type or heterozygous *igf2bp3*^*la659Tg/+*^) embryos showed weak expression only from sphere stages (Fig 3A and 3B). Similarly, a sqt-gfp reporter lacking the binding motif for Ybx1 (ΔYBE Sqt-GFP) also showed elevated expression in MZ*igf2bp3*^*la659Tg*^ embryos (S5A and S5B Fig). Therefore, maternal Igf2bp3 likely functions in translational control of Nodal signaling in early zebrafish embryos.

### Igf2bp3 is essential for proper germline development

**Fig 4:**
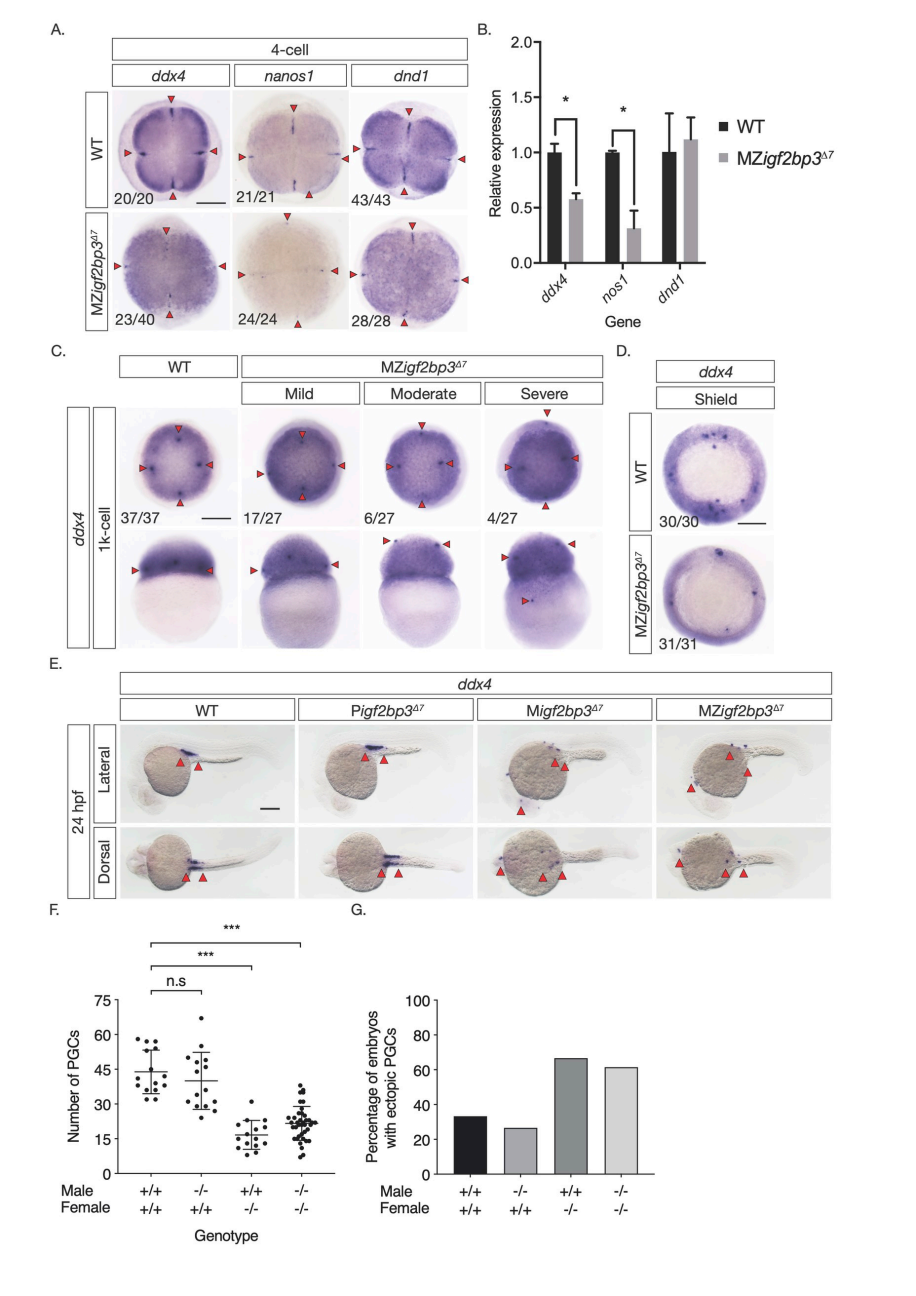
Igf2bp3 is required for normal germline development. A. Whole mount in-situ hybridisation (WISH) in early embryos shows altered expression of germline markers *ddx4*, *dnd1*, and *nanos1* in *igf2bp3*^*Δ7*^ mutant embryos. B. qRT-PCR to detect early germplasm markers shows reduced levels of *ddx4* and *nos1* expression in mutant embryos at the 4-cell stage, whereas expression levels of *dnd1* is not significantly different from control wild type embryos. C. Primordial germ cells (PGCs; red arrowheads) in *igf2bp3*^*Δ7*^ embryos are ectopically located at the 1k-cell stage to varying extents ranging from mild or moderate to severe. D. Primordial germ cells are severely reduced or not detected in *igf2bp3*^*Δ7*^ mutants by gastrula stages. E,F. WISH (E) and quantitation (F) of *ddx4*-positive cells (red arrowheads) shows reduced and ectopic primordial germ cells in 24 hpf maternal *igf2bp3*^*Δ7*^ (M*igf2bp3*^*Δ7*^) and maternal-zygotic *igf2bp3*^*Δ7*^ mutants (MZ*igf2bp3*^*Δ7*^) compared to WT siblings and paternal *igf2bp3*^*Δ7*^ (P*igf2bp3*^*Δ7*^) mutants; p *<0.05, **<0.01, ***< 0.001. G. Loss of maternal *igf2bp3* leads to some ectopic primordial germ cells across the trunk (red arrowheads) and occasionally in the hindbrain region. Bar graph shows the number of embryos with ectopic germ cells in WT, P*igf2bp3*^*Δ7*^, M*igf2bp3*^*Δ7*,^ and MZ*igf2bp3*^*Δ7*^ mutants. Scale bar in A and C-E, 200 μm. N = 15 embryos each for WT, P*igf2bp3*^*Δ7*^, and M*igf2bp3*^*Δ7*^ and 39 for MZ*igf2bp3*^*Δ7*^ mutants.

We found altered distribution of *dazl* RNA in MZ*igf2bp3*^Δ7^ mutant embryos at the 1-cell stage, and altered sex ratios with a male bias in Z*igf2bp3*^Δ7^ as well as MZ*igf2bp3*^*la659Tg*^ adults. In zebrafish, male sex bias can arise from depletion of germplasm or primordial germ cells (PGCs). Therefore, we examined the expression of *ddx4*, *nos1* and *dnd1*, in MZ*igf2bp3*^*Δ7*^ embryos. WISH and qRT-PCRs show that germplasm markers are reduced in mutant embryos from the 4-cell stage (Fig 4A and 4B). PGCs are displaced and found in ectopic positions in the blastoderm from the 1K-cell stage and reduced during gastrulation (Fig 4C and 4D). Ectopic and reduced number of PGCs are observed in the gonadal ridge by 24 hpf (Fig 4E–4G) in maternal *igf2bp3* mutants (progeny of wild-type males crossed with mutant females, M*igf2bp3*) and MZ*igf2bp3* mutants, but not in wild type (WT) and paternal *igf2bp3* mutants (progeny of *igf2bp3* males crossed with WT females, P*igf2bp3*). These defects are also observed in MZ*igf2bp3*^*la361Tg*^ and MZ*igf2bp3*^*la659Tg*^ mutant embryos (S6A–S6G Fig). The reduction in PGCs is not rescued by injection of either *igf2bp1* or *igf2bp3* capped mRNA (S4B Fig). Therefore, maternal Igf2bp3 function is crucial for normal PGC development.

### Germline progenitor cells mis-migrate in MZ*igf2bp3* embryos

**Fig 5:**
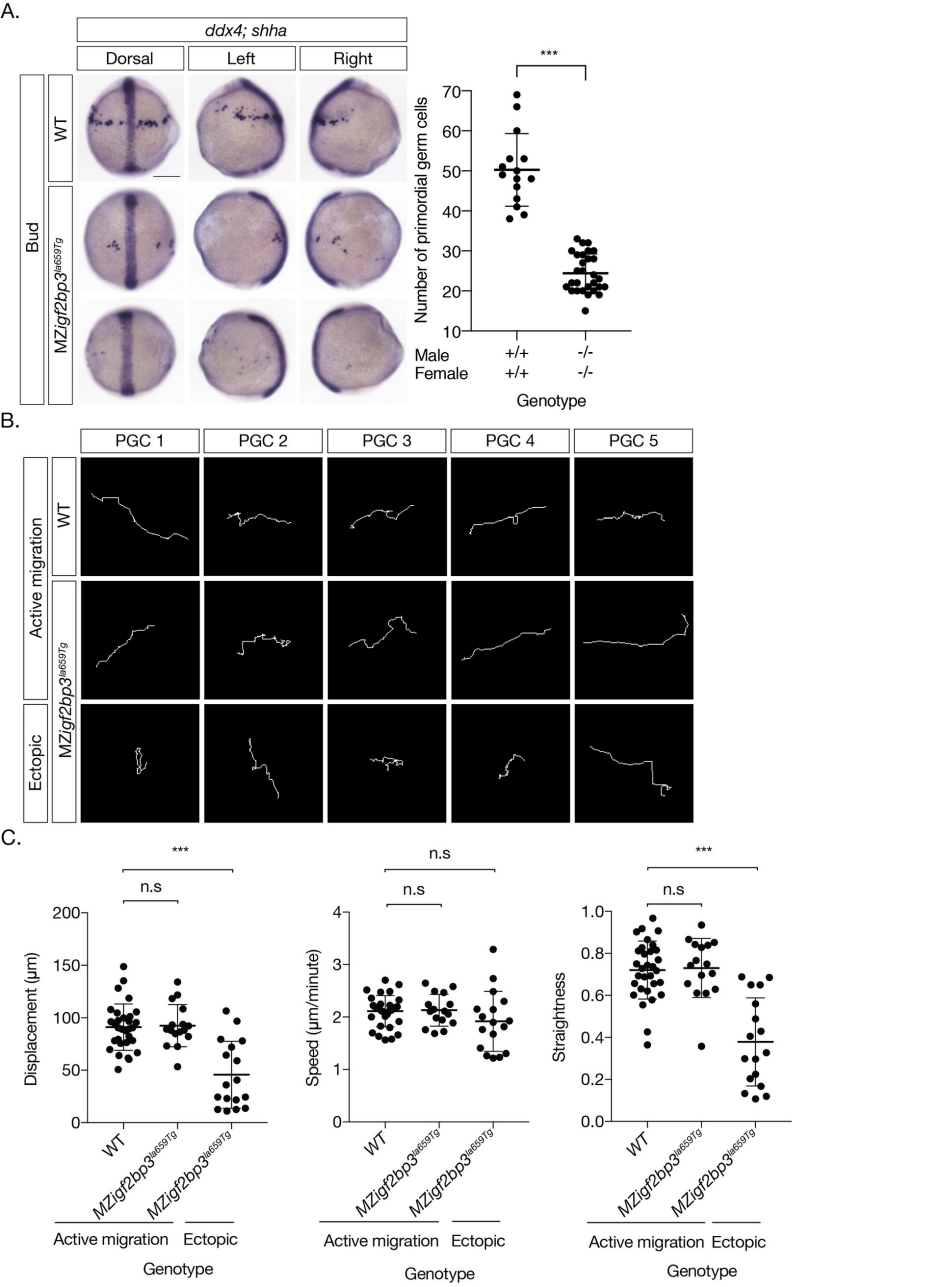
Primordial germ cell migration is aberrant in *igf2bp3* mutant embryos. A. WISH to detect *ddx4* positive PGCs and *shh* in the midline at Bud stage. PGCs are reduced (scatter plot, right) and ectopically located in MZ*igf2bp3*^*la659Tg*^ embryos compared to wild type (WT) embryos. B. Migration tracks of PGCs in bud stage embryos, labelled with *GFP-nos1* reporter. MZ*igf2bp3*^*la659Tg*^ embryos show aberrant PGC migration compared to WT controls. C. PGC track analysis showing displacement, speed and straightness index. Ectopic PGCs in MZ*igf2bp3*^*la659Tg*^ mutants show significantly reduced total displacement and straightness, although speed of individual PGCs is not altered in mutant embryos compared to WT controls. Scale bars, 200 μm; p*<0.05, **<0.01, ***<0.001; Number of PGCs analysed = 31, 16 and 17 for WT, actively migrating and ectopic MZ*igf2bp3*^*la659Tg*^ PGCs, respectively.

To understand the basis of the PGC defects in *igf2bp3* mutant embryos, we performed live-imaging of PGCs using a GFP-nos1 3’UTR fluorescent reporter. We detected aberrant and convoluted PGC migration in MZ*igf2bp3*^*la659Tg*^ embryos from late-gastrula stage (S1 Movie), and PGCs are spread out along the animal-vegetal axis in mutant embryos. At the tailbud stage (10 hpf), both control and MZ*igf2bp3*^*la659Tg*^ embryos show trailing clusters of PGCs (S2 and S3 Movies). However, PGC numbers are reduced in MZ*igf2bp3*^*la659Tg*^ (Fig 5A and 5B) and *igf2bp3*^*Δ7*^ (S7A and S7B Fig) mutant embryos. Many PGCs are found at ectopic locations, with some even in the hindbrain region (Figs 4E and S6C and S6F) in mutant embryos and a number of PGCs significantly further away from the midline (S3 Movie). Some mutant PGCs form clusters that are not closely associated with other PGCs, indicating disruptions in collective migration of mutant germline cells.

We compared the behaviour of actively migrating cells and ectopic PGCs in WT and *igf2bp3*^*la659Tg*^ embryos. PGC migration tracks were traced and movement dynamics (speed, displacement and track straightness) were measured. Actively migrating PGCs exhibit similar dynamics in mutant and wild type embryos and produce linear tracks (Fig 5B). By contrast, ectopic PGCs in *igf2bp3* mutant embryos show significantly convoluted paths, resulting in reduced net displacement over the course of migration (Fig 5C and S4 Movie).

**Fig 6:**
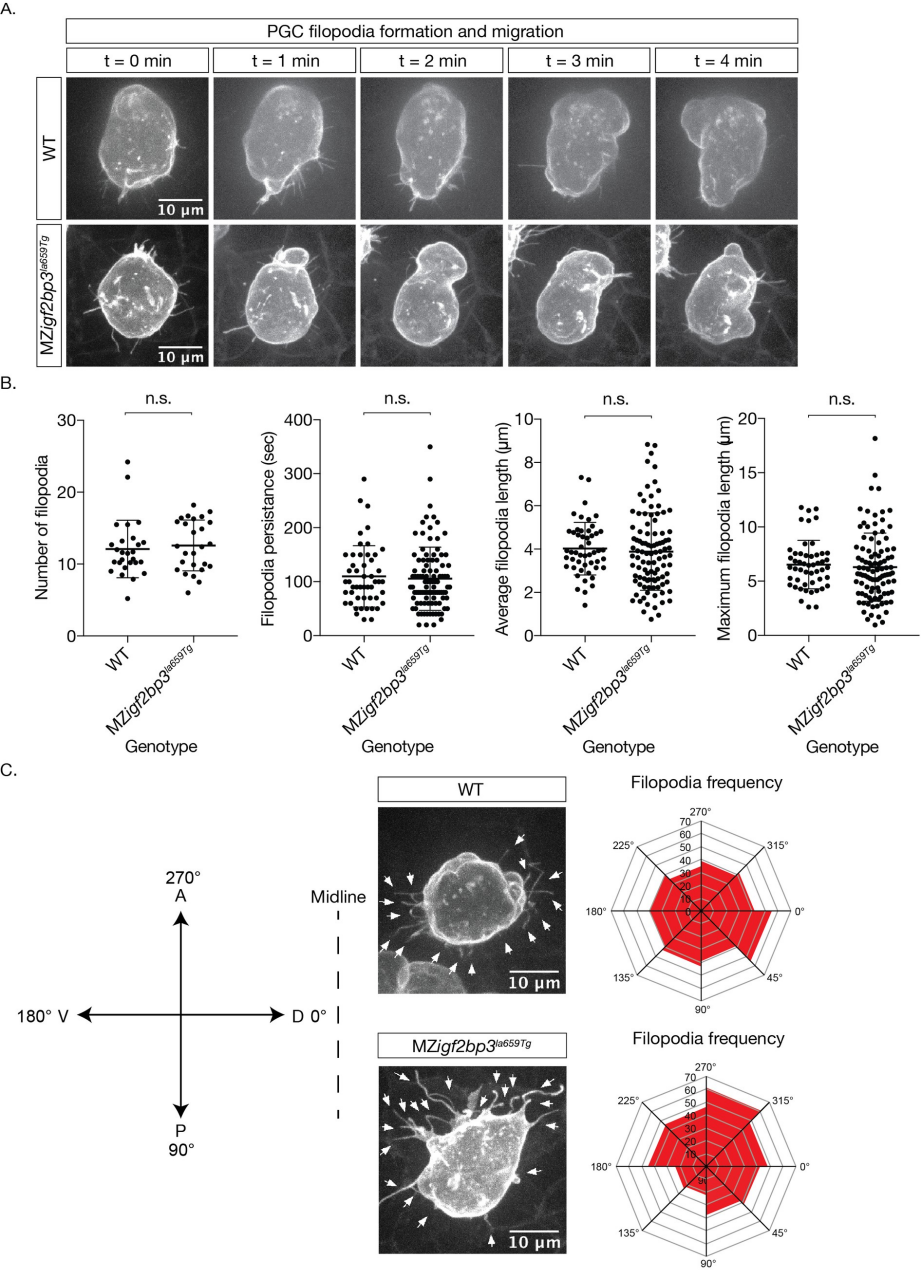
PGC membrane projections show altered behaviour in MZ*igf2bp3*^*la659Tg*^ mutants. A. Images of cell membranes of PGCs labelled with a Farnesylated-*GFP-nos*1 live reporter in bud stage mutant or wild type embryos. B. Quantitation of filopodia and behaviour (persistence, length) in WT and MZ*igf2bp3*^*la659Tg*^ PGCs. C. Projection angle relative to their destination, showing an altered skew in maternal *igf2bp3*^*la659Tg*^ PGCs, although the projection parameters, namely numbers, persistence, average and maximum lengths did not appear to be changed. p *<0.05, **<0.01, ***<0.001. N = 27 WT and 26 MZ*igf2bp3*^*la659Tg*^ PGCs; number of filopodia = 50 WT and 100 mutant filopodia. Number of PGCs analysed for projection frequency = 25 per genotype; Scale bars, 10 μm.

In order to investigate further the aberrant migration in *igf2bp3* mutants, we analysed the behaviour of individual PGCs and imaged the actin-based protrusions, filopodia. We injected farnesylated eGFP mRNA to label the PGCs, and imaged embryos from 10 hpf. The behaviour of individual filopodia was measured over their lifetime: the number of filopodia per PGC, the persistence of each filopodium, the average and maximum length. The overall morphology of the PGCs appeared to be consistent (Fig 6A), and the number and length of the filopodia were comparable through the duration of time-lapse imaging (Fig 6B). In WT PGCs, the projections are biased towards the midline, shown as an increase of filopodia with projections towards the right (S5 Movie). However, in MZ*igf2bp3*^*la659Tg*^ PGCs, the filopodial projections are not directed towards the midline. (Fig 6C and S6 Movie). We also observed extensive blebbing, but without any particular orientation in mutant PGCs (S6 Movie). Therefore, PGC behaviour is perturbed in *igf2bp3*^*la659Tg*^ mutant embryos.

### MZ*igf2bp3* mutants have defects in the chemokine guidance system

PGC migration and cell behaviours depend on the chemokine guidance system, and disruptions in chemokine signalling can lead to aberrant PGC behaviours and mis-migration [60]. We examined the expression of chemokine guidance system components in MZ*igf2bp3*^*la659Tg*^ and MZ*igf2bp3*^*Δ7*^ embryos. We find a significant increase in expression of the chemokine receptors *cxcr4a* and *cxcr4b* in both mutants at the 1K-cell stage, and during gastrulation, levels of the chemokine ligand *cxcl12a* are significantly reduced (S8 Fig). Therefore, the PGC migration defects and aberrant cell behaviours in maternal *igf2bp3* mutant PGCs likely arise from defects in the chemokine guidance system.

### Mis-migrating PGCs in MZ*igf2bp3* mutants undergo cell death

**Fig 7:**
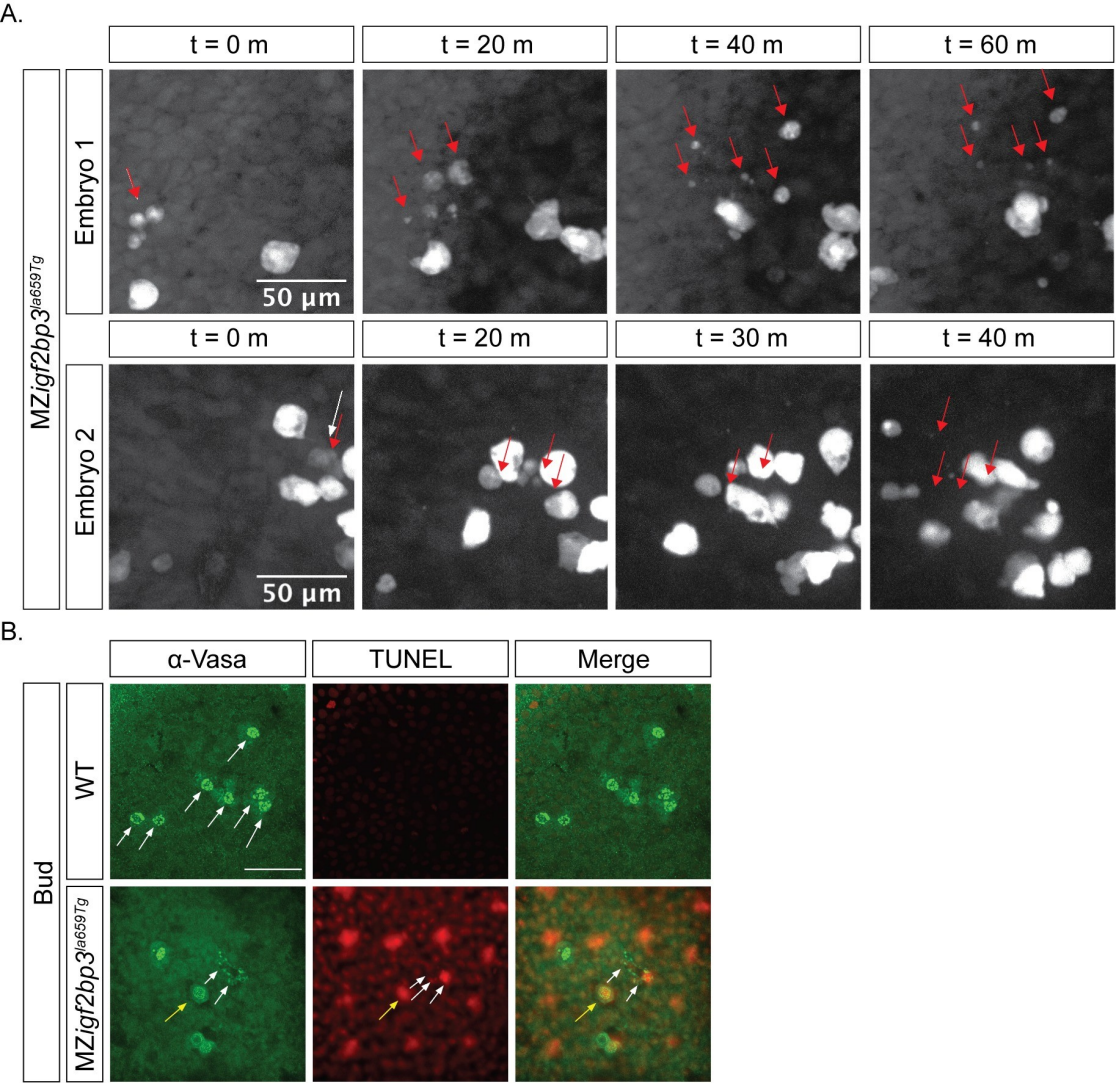
Loss of PGCs in MZ*igf2bp3*^*la659Tg*^ mutant embryos might arise from cell death. A. Some PGCs are lost during migration in maternal *igf2bp3*^*la659Tg*^ embryos. At bud stage, a subset of PGCs labelled with a *GFP-nos1* live reporter display fragmentation during migration. B. Immunofluorescence with an α-Vasa antibody and TUNEL staining showed that compared to wild type embryos, in MZ*igf2bp3*^*la659Tg*^ embryos a subset of PGCs undergo cell death and fragmentation at the bud stage. Yellow arrow indicates co-localisation of Vasa and TUNEL labelling in a PGC, white arrows indicate Vasa+ fragments; Scale bar, 50 μm.

Finally, to investigate the reduction in PGC numbers in mutant embryos, we examined if cell proliferation and/or cell death are altered. Using live reporters, we did not observe any defects in PGC cell divisions in mutant embryos at gastrula or tailbud stages. However, some MZ*igf2bp3*^*la659Tg*^ PGCs fragment along their migration path from the tailbud stage (Fig 7A and S7 and S8 Movies). TUNEL labelling on fixed tailbud stage MZ*igf2bp3*^*la659Tg*^ mutant or wild type embryos together with immunostaining for Vasa protein shows Vasa-positive fragments, and co-localisation of the TUNEL label with Vasa-positive PGCs is observed in some cells (Fig 7B). Fragmentation is seen in 14% of mutant PGCs at tailbud (7/49 Vasa+ cells, n = 11 MZ*igf2bp3* embryos), whereas no fragmentation of Vasa-positive cells was found in wild type embryos (0/83 Vasa+ cells, n = 8 WT embryos). Therefore, PGC survival is compromised in the absence of Igf2bp3 function.

## Discussion

In this study, we examined the role of the conserved RNA-binding protein, Igf2bp3, in zebrafish, and found that Igf2bp3 is essential for germline development and normal sex ratios in adults, suggesting that Igf2bp3 plays crucial roles in sex determination. We also uncovered a function for Igf2bp3 in early embryonic development, and specifically in the allocation of embryonic versus extra-embryonic cell fates, with maternal mutant embryos showing expansion of the YSL at the expense of the embryonic blastoderm. The YSL expansion, embryonic lethality and germline defects are not rescued by injection of *igf2bp3* RNA into mutant embryos. Therefore, maternal Igf2bp3 is likely to play crucial roles during normal embryonic and germline development.

In a small number of *igf2bp3* deletion mutant embryos, we also found cell adhesion defects and these embryos typically do not survive beyond gastrula stages. The cell adhesion defects are similar to the phenotypes described for a different *igf2bp3* mutant allele [34]. The majority of our *igf2bp3* deletion mutant embryos show expansion of the YSL and we found enhanced expression of the early YSL marker, *mxtx2*. By contrast, the expression of *sqt/nodal* and *ntl/tbxta* is variably increased in *igf2bp3* mutant embryos at the 1K cell stage, and either reduced or comparable to control levels by early gastrula stages. Interestingly, we observed elevated levels of the mesendoderm gene *gsc*, which is a target of Nodal signalling. Consistent with this observation, we found premature and elevated levels of a Nodal reporter. This suggests premature translation and de-regulated Nodal signalling in maternal *igf2bp3* mutant embryos. We previously showed that the RNA-binding protein Ybx1 (which was identified in an RNP complex together with Igf2bp3), also functions in translational repression of Nodal signalling in early zebrafish embryos [21]. Interestingly, a Nodal reporter lacking the binding motif for Ybx1 also showed elevated expression in MZ*igf2bp3* embryos, indicating that translational control by Igf2bp3 is likely independent of binding by Ybx1.

Previous studies in zebrafish and *Xenopus* found that *igf2bp3* transcript is enriched in the Balbiani body [20], a transient structure in oocyte development, where germplasm is initially aggregated, and a recent report showed that Igf2bp3 protein interacts with the germplasm protein Bucky ball in yeast-two hybrid assays and through co-immunofluorescence [61]. The presence of Igf2bp3 as an RNA-binding protein in this structure suggests that it might regulate many RNAs involved in germline development. Accordingly, we find that germline transcripts including *ddx4*, *nanos1* and *dnd1*, are reduced in maternal *igf2bp3* mutant embryos at early stages. Recent studies examining the zebrafish transcriptome in *igf2bp3* mutants reported that *igf2bp3* acts as a stability factor for maternal and germplasm RNAs in zebrafish [34,61]. We have observed additional phenotypes that have not previously been reported, such as a mis-migration of germ cells and male sex bias in adult zygotic *igf2bp3* mutants. Therefore, *igf2bp3* might have crucial roles in further processes that are not strictly maternal.

Some germplasm RNAs are critical for the survival, proliferation or migration of PGCs. For example, *dnd1* RNA acts as a survival and migration factor for zebrafish PGCs [16]. We found a reduction of germline RNAs, which is consistent with the reduced number of PGCs in *igf2bp3* mutants at gastrula stages. In addition, we also observed ectopic germ cells at mid-late blastula stages. It is possible that the expanded YSL and reduced blastoderm results in altered positioning of germ cells in *igf2bp3* mutant blastulae. This might be exacerbated by abnormal chemokine guidance system, leading to abnormal migratory behavior during gastrula and somitogenesis stages, resulting in ectopic localisation and depletion of PGCs. We also observed increased frequency of blebbing in mutant PGCs, but without any particular orientation. Increased blebs can be found associated with apoptosis [62], and consistently, we found that a proportion of mutant PGCs undergo cell death during somitogenesis. Together, the initial loss of germplasm RNAs combined with altered blastoderm and germ cell position, and defects in chemokine guidance likely results in the progressive reduction and eventual loss of PGCs in *igf2bp3* mutant embryos. Early loss of PGCs is known to promote testes formation [63] and might underlie the male bias found in *igf2bp3* mutant adults.

The mechanism through which Igf2bp3 acts to regulate the germplasm and the germline remains unknown. The mechanisms that determine sex in zebrafish are also not understood [64]. Many proteins associated with m6A modifications, known as ‘m6A readers’, have been shown to be important for fertility and germline development, such as Ythdf2 [31], Ythdc2 [30,65,66] and a closely related Drosophila homolog, Bcgn [67]. Igf2bp proteins were found to be associated with m6A modifications [32]. Our findings, showing an essential role for Igf2bp3 in embryonic versus extraembryonic and germline progenitors in zebrafish, are consistent with these reports, and identifies Igf2bp3 as part of a potentially conserved network of proteins involved in RNA regulation in early embryos and in the germline.

## Supporting information

S1 Fig *igf2bp3* is expressed ubiquitously during early development. A. RNA-seq expression data for all four zebrafish *igf2bp* genes during zebrafish development shows that *igf2bp3* is expressed throughout zebrafish development. Data from [68]. B. Whole mount *in-situ* hybridisation (WISH) to detect *igf2bp3* in zebrafish embryos at cleavage, blastula, gastrula, somitogenesis and 24 hpf. C. Sex ratios in *igf2bp3*^*Δ7/+*,^ *igf2bp3*^*Δ7/Δ7*^, *igf2bp3*^*la659Tg/+*^, and *igf2bp3*^*la659Tg/la659Tg*^ (i.e. zygotic *igf2bp3*^*la659Tg*^) adult fish harbouring the *Tg(buc*:*buc-eGFP)* transgene show that transgenic zygotic *igf2bp3*^*Δ7/Δ7*^ mutants manifest a strong male bias. Scale bar in B, 200 μm.
(TIF)

S2 Fig Maternal *igf2bp3*^*la659Tg*^ mutant embryos show no detectable *igf2bp3* transcript or Igf2bp3 protein. A. Schematic representation of the *igf2bp3*^*la659Tg*^ mutant locus showing the site of the 6 kb *Tg(nlacZ-GT virus)* gene trap retroviral insertion, and coloured triangles indicate the position of RT-PCR primers. B,C. MZ*igf2bp3*^*la659Tg*^ mutant embryos show significantly reduced or undetectable expression of *igf2bp3*. RT-PCR (gels on left) using exon spanning primers for *igf2bp3* and qRT-PCR (bar graphs, right) at performed at six early developmental stages, show reduced *igf2bp3* transcripts in mutant embryos relative to *gapdh* control. C. Igf2bp3 protein is not detectable in MZ*igf2bp3*^*la659Tg*^ embryos. Western blots on cleavage to early gastrula stage embryos show a 60 kD band in wild type (WT) lysates. No Igf2bp3 band is detected in MZ*igf2bp3*^*la659Tg*^ lysates. D. WISH shows that *shh* expression in the midline (top panels) and *myoD* expression in the myotome (bottom panels) in MZ*igf2bp3*^*la659Tg*^ mutants is similar to wild type (WT) controls. E. Images of live five-day larvae show that the swim bladder inflates normally in *igf2bp3*^*la659Tg/+*^, zygotic *igf2bp3*^*la659Tg*^ and MZ*igf2bp3*^*la659Tg*^ mutants, and there are no overt morphological defects in mutant larvae. F. Early cell divisions and cytokinesis are similar in WT and MZ*igf2bp3*^*la659Tg*^ mutant embryos. G. *igf2bp* family genes are not significantly altered in MZ*igf2bp3*^*la659Tg*^ mutants. Expression of *igf2bp1*, *igf2bp2a* (not expressed prior up to 1K-cell) and *igf2bp2b* were measured at 1-cell, 1K-cell and 50%-epiboly in WT and MZ*igf2bp3*^*la659Tg*^ mutants, revealing no changes in expression. Scale bars in D,E, 200 μm.
(TIF)

S3 Fig The extra-embryonic yolk syncytial layer (YSL) is transiently expanded in maternal *igf2bp3*^*la659Tg*^ mutant embryos. A. Immunofluorescence to detect membrane β-Catenin (green) and nuclear DAPI (blue) in wild type and MZ*igf2bp3*^*la659Tg*^ mutant embryos at 3 hpf (left) or 4.5 hpf (right) shows expanded YSL in mutant embryos at 3 hpf but not at 4.5 hpf. B. The distance of the farthest yolk syncytial nuclei (YSN) from the blastoderm margin (dashed yellow lines in embryos shown in A) and the number of YSN in WT and MZ*igf2bp3*^*la659Tg*^ mutant embryos show an initial increase at 3 hpf in mutant embryos, but becomes similar to WT at 4.5 hpf. N = 20 for both genotypes at 3 hpf and 4.5 hpf; statistical analysis performed with two-tailed unpaired t-test; p * < 0.05, ** < 0.01, *** < 0.001. C-E. qRT-PCR in 1-cell (C), 1000-cell (1K; D) and 50% epiboly stage (E) wild type and MZ*igf2bp3*^*la659Tg*^ mutant embryos shows significantly reduced *igf2bp3* transcript levels in mutant embryos at all stages; *mxtx2* is not altered at 1-cell and 1K stages but reduced at 50% epiboly in MZ*igf2bp3*^*la659Tg*^ mutants, and *camsap3* is either unchanged (1-cell and 1K), or slightly increased (50% epiboly) in MZ*igf2bp3*^*la659Tg*^; p *** < 0.01, **** <0.001. Scale bar in A, 200 μm.
(TIF)

S4 Fig Injection of *igf2bp3* mRNA does not rescue maternal *igf2bp3* phenotypes. A. Injection of *igf2bp3* mRNA into *igf2bp3*^*Δ7*^ embryos does not rescue YSL expansion or lethality. WT, M*igf2bp3*^*Δ7*^ and MZ*igf2bp3*^*Δ7*^ embryos were injected with 200 pg of *igf2bp3* mRNA at the 1-cell stage and the YSL phenotype scored at 3 hpf. B. Injection of *igf2bp1* or *igf2bp3* mRNA into *igf2bp3*^*la659Tg*^ does not rescue loss of PGCs. MZ*igf2bp3*^*la659Tg*^ embryos were injected with 100 pg of *igf2bp1* or *igf2bp3* mRNA at the 1-cell stage and the number of PGCs quantified at 24 hpf.
(TIF)

S5 Fig Maternal *igf2bp3* mutants show premature and elevated expression of a Nodal translation reporter lacking a 3’UTR Ybx1 binding motif. A. ΔYBE Sqt-GFP reporter expression (green signals in extracellular space) is premature and elevated in MZ*igf2bp3*^*la659Tg*^ embryos compared to control embryos at the high, sphere and dome stages. B. Bar graphs show mean GFP signal intensity in the blastoderm of imaged embryos. GFP levels in the blastoderm were normalised to levels of co-injected rhodamine dextran control. Representative examples from three independent experiments are shown. Number of embryos analysed: high (N = 3 WT and 4 MZ*igf2bp3*^*la569Tg*^), sphere (N = 6 control and 7 MZ*igf2bp3*^*la569Tg*^), dome (N = 4 WT and 5 MZ*igf2bp3*^*la569Tg*^). Asterisks indicate level of significance from two-tailed t tests; p *<0.05, **<0.01, ***<0.001. Scale bar, 50 μm.
(TIF)

S6 Fig The germline is mis-regulated in *igf2bp3* insertion mutants. A,B. Live imaging of PGCs in embryos injected with GFP-*nos*3’UTR reporter mRNA shows reduced PGC numbers in *igf2bp3*^*la659Tg*^ mutants compared to WT embryos at 1-somite, 5-somite, 10-somites and 25-somites. C-E. PGC numbers are reduced and germ cells are ectopically located in MZ*igf2bp3*^*la659Tg*^ mutants (C, E), and reduced in both M*igf2bp3*^*la659*Tg^ and MZ*igf2bp3*^*la659*Tg^ embryos (D). F. The germline is also mis-regulated in a second transgenic insertion mutant line, *igf2bp3*^*la361Tg*^ with significantly reduced PGCs in MZ*igf2bp3*^*la361Tg*^ embryos (F, G). N = 15 WT, 20 P*igf2bp3*^*la659Tg*^ 20 M*igf2bp3*^*la659Tg*^ and 25 MZ*igf2bp3*^*la659Tg*^ embryos in C-E, and 5 WT and 23 MZ*igf2bp3*^*la361Tg*^ mutants in F,G. Scale bars in C and F, 200 μm; * p<0.05, **<0.01, ***<0.001.
(TIF)

S7 Fig PGC migration is aberrant and numbers are reduced in maternal *igf2bp3*^*Δ7*^ mutant embryos. A,B. WISH to detect the germline marker *ddx4* (A) shows that PGCs are reduced (quantitation in B), and ectopically located relative to the midline (dashed line) in M*igf2bp3*^*Δ7*^ embryos by Bud stage. N = 39 WT and 42 MZ*igf2bp3*^*Δ7*^ mutants; Scale bar in A, 200 μm; p* < 0.05, ** < 0.01, *** < 0.001.
(TIF)

S8 Fig Expression of germline and mesendoderm RNAs is altered in maternal *igf2bp3* mutants. A-F. qRT-PCR of M*igf2bp3*^*Δ7*^, MZ*igf2bp3*^*Δ7*^ or MZ*igf2bp3*^*Δ7*^ in a *Tg(buc*:*buc-egfp)*; *buc*^*p106+/-*^ background and MZ*igf2bp3*^*la659Tg*^ (G-K) mutant embryos compared to wildtype controls, shows variable reduction in *nos1*, *ddx4*, *dnd1* and *dazl* expression in one-cell stage mutant embryos. Reduction in *ddx4*, *nos1*, *dnd1* and *gran* is observed in MZ*igf2bp3*^*Δ7*^ and MZ*igf2bp3*^*la659Tg*^ embryos at the 1K stage. C, H. At the 1K stage, expression of the chemokine receptors, *cxcr4a* and *cxcr4b*, is elevated in *igf2bp3* mutant embryos, whereas at 50% epiboly (E, J) *ddx4* and *cxcl12a* levels are reduced in the mutants. At 1K-cell stage, there is an increase in *sqt* and a variable increase in *tbxta* in both mutants (D, I). At 50% epiboly (F, K), *sqt* is significantly reduced or reverts to normal levels in MZ*igf2bp3*^*la659Tg*^ and MZ*igf2bp3*^*Δ7*^ embryos, respectively; *gsc* is significantly increased in both mutants. Data from 3 biological replicates of 25–50 embryos each; p *<0.09, **<0.05, ***<0.01, ****<0.001.
(TIF)

S1 Movie MZ*igf2bp3*^*la659Tg*^ embryos show convoluted PGC migration paths from gastrula stages. GFP-labelled PGCs in a late gastrula (80% epiboly) *MZigf2bp3*^*la659Tg*^ embryo show convoluted migration paths and considerable spread along the animal-vegetal axis. In the clip shown, dorsal is to the right, animal pole towards the top and the gastrula margin to the bottom.
(AVI)

S2 Movie WT PGCs migrate towards the midline during Bud stage. PGCs expressing GFP in 10 hpf WT embryos tracked with spinning disk confocal microscopy. Cells cluster together and migrating rapidly towards the midline (red dashed line).
(AVI)

S3 Movie MZ*igf2bp3*^*la659Tg*^ mutant PGCs are reduced during Bud stage. PGCs expressing GFP in 10 hpf mutant embryos, imaged with spinning disk confocal microscopy. Reduced number of cells cluster and migrate towards the midline in the mutant.
(AVI)

S4 Movie Ectopic MZ*igf2bp3*^*la659Tg*^ mutant PGCs migrate anomalously. Ectopic and dispersed PGCs in an MZ*igf2bp3*^*la659Tg*^ mutant embryo at bud stage. The PGCs move in a non-linear fashion.
(AVI)

S5 Movie WT PGCs extend filopodia in a directed manner towards the midline. PGCs in a 10 hpf WT embryo, expressing Farnesylated-eGFP, tracked with spinning disk confocal microscopy, showing cellular projections towards the midline (right, indicated by a white arrow).
(AVI)

S6 Movie MZ*igf2bp3*^la659Tg^ mutant PGCs produce filopodia with altered directionality. PGCs expressing Farnesylated-eGFP in a MZ*igf2bp3*^*la659Tg*^ embryo tracked with spinning disk confocal microscopy. Extensive blebbing is observed and projections are not directed towards the midline (right) in the mutant embryo.
(AVI)

S7 Movie MZ*igf2bp3*^*la659Tg*^ mutant PGCs exhibit fragmentation during migration. PGCs expressing GFP in MZ*igf2bp3*^*la659Tg*^ embryos tracked with spinning disk confocal microscopy. One PGC fragments (red arrow) and scatters during migration towards the midline (dashed red line, left) in the mutant embryo.
(AVI)

S8 Movie MZ*igf2bp3*^*la659Tg*^ mutant PGCs exhibit fragmentation during migration. PGCs expressing GFP in MZ*igf2bp3*^*la659Tg*^ embryos tracked with spinning disk confocal microscopy. One PGC fragments (red arrow) and scatters during its migration towards the midline (dashed red line, right) in the mutant.
(AVI)

S1 Methods List of primers and aptamer sequences. Primers for qRT-PCR, genotyping, and sequence of aptamers for tobramycin affinity pull-downs.
(DOCX)
