# Supplementary figures and images for: The RNA-binding protein Igf2bp3 is critical for embryonic and germline development in zebrafish
Source: PLoS Genet. 2021 Jul 2;17(7):e1009667. doi: 10.1371/journal.pgen.1009667 (PMC8282044; doi:10.1371/journal.pgen.1009667)

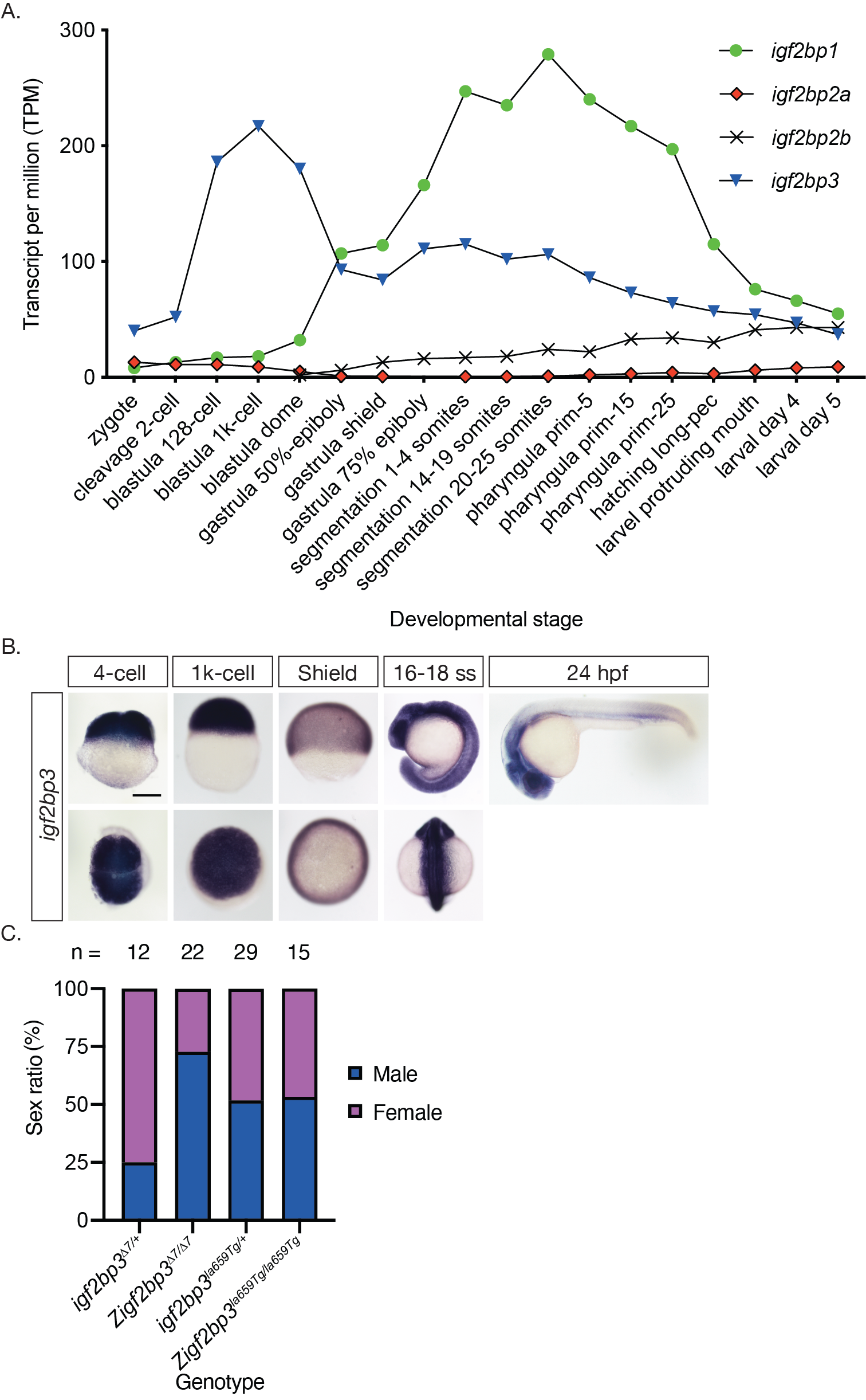

Supplement: S1 Fig — A. RNA-seq expression data for all four zebrafish igf2bp genes during zebrafish development shows that igf2bp3 is expressed throughout zebrafish development. Data from [68]. B. Whole mount in-situ hybridisation (WISH) to detect igf2bp3 in zebrafish embryos at cleavage, blastula, gastrula, somitogenesis and 24 hpf. C. Sex ratios in igf2bp3Δ7/+, igf2bp3Δ7/Δ7, igf2bp3la659Tg/+, and igf2bp3la659Tg/la659Tg (i.e. zygotic igf2bp3la659Tg) adult fish harbouring the Tg(buc:buc-eGFP) transgene show that transgenic zygotic igf2bp3Δ7/Δ7 mutants manifest a strong male bias. Scale bar in B, 200 μm. (TIF) [file pgen.1009667.s001.tif]

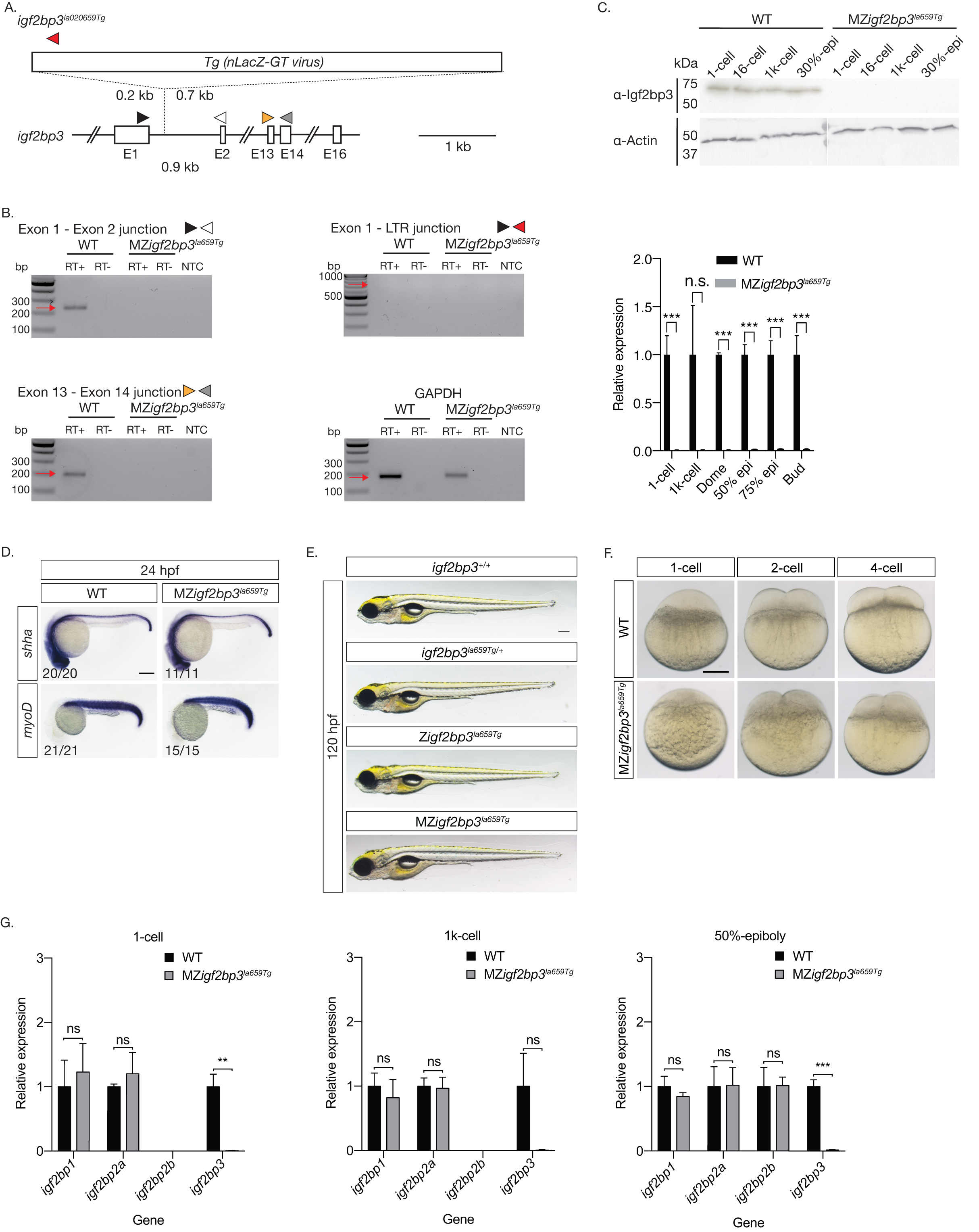

Supplement: S2 Fig — A. Schematic representation of the igf2bp3la659Tg mutant locus showing the site of the 6 kb Tg(nlacZ-GT virus) gene trap retroviral insertion, and coloured triangles indicate the position of RT-PCR primers. B,C. MZigf2bp3la659Tg mutant embryos show significantly reduced or undetectable expression of igf2bp3. RT-PCR (gels on left) using exon spanning primers for igf2bp3 and qRT-PCR (bar graphs, right) at performed at six early developmental stages, show reduced igf2bp3 transcripts in mutant embryos relative to gapdh control. C. Igf2bp3 protein is not detectable in MZigf2bp3la659Tg embryos. Western blots on cleavage to early gastrula stage embryos show a 60 kD band in wild type (WT) lysates. No Igf2bp3 band is detected in MZigf2bp3la659Tg lysates. D. WISH shows that shh expression in the midline (top panels) and myoD expression in the myotome (bottom panels) in MZigf2bp3la659Tg mutants is similar to wild type (WT) controls. E. Images of live five-day larvae show that the swim bladder inflates normally in igf2bp3la659Tg/+, zygotic igf2bp3la659Tg and MZigf2bp3la659Tg mutants, and there are no overt morphological defects in mutant larvae. F. Early cell divisions and cytokinesis are similar in WT and MZigf2bp3la659Tg mutant embryos. G. igf2bp family genes are not significantly altered in MZigf2bp3la659Tg mutants. Expression of igf2bp1, igf2bp2a (not expressed prior up to 1K-cell) and igf2bp2b were measured at 1-cell, 1K-cell and 50%-epiboly in WT and MZigf2bp3la659Tg mutants, revealing no changes in expression. Scale bars in D,E, 200 μm. (TIF) [file pgen.1009667.s002.tif]

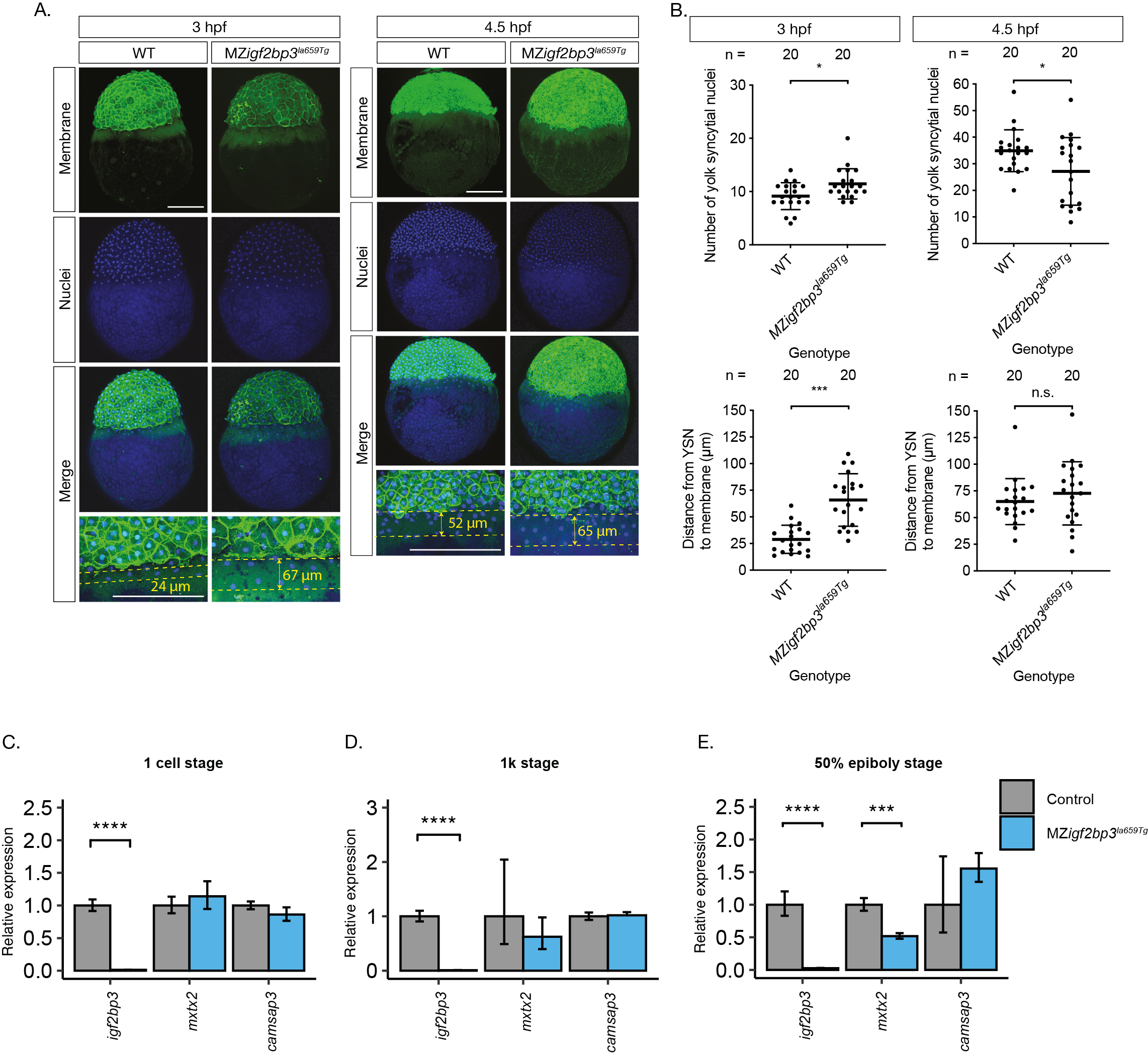

Supplement: S3 Fig — A. Immunofluorescence to detect membrane β-Catenin (green) and nuclear DAPI (blue) in wild type and MZigf2bp3la659Tg mutant embryos at 3 hpf (left) or 4.5 hpf (right) shows expanded YSL in mutant embryos at 3 hpf but not at 4.5 hpf. B. The distance of the farthest yolk syncytial nuclei (YSN) from the blastoderm margin (dashed yellow lines in embryos shown in A) and the number of YSN in WT and MZigf2bp3la659Tg mutant embryos show an initial increase at 3 hpf in mutant embryos, but becomes similar to WT at 4.5 hpf. N = 20 for both genotypes at 3 hpf and 4.5 hpf; statistical analysis performed with two-tailed unpaired t-test; p * < 0.05, ** < 0.01, *** < 0.001. C-E. qRT-PCR in 1-cell (C), 1000-cell (1K; D) and 50% epiboly stage (E) wild type and MZigf2bp3la659Tg mutant embryos shows significantly reduced igf2bp3 transcript levels in mutant embryos at all stages; mxtx2 is not altered at 1-cell and 1K stages but reduced at 50% epiboly in MZigf2bp3la659Tg mutants, and camsap3 is either unchanged (1-cell and 1K), or slightly increased (50% epiboly) in MZigf2bp3la659Tg; p *** < 0.01, **** <0.001. Scale bar in A, 200 μm. (TIF) [file pgen.1009667.s003.tif]

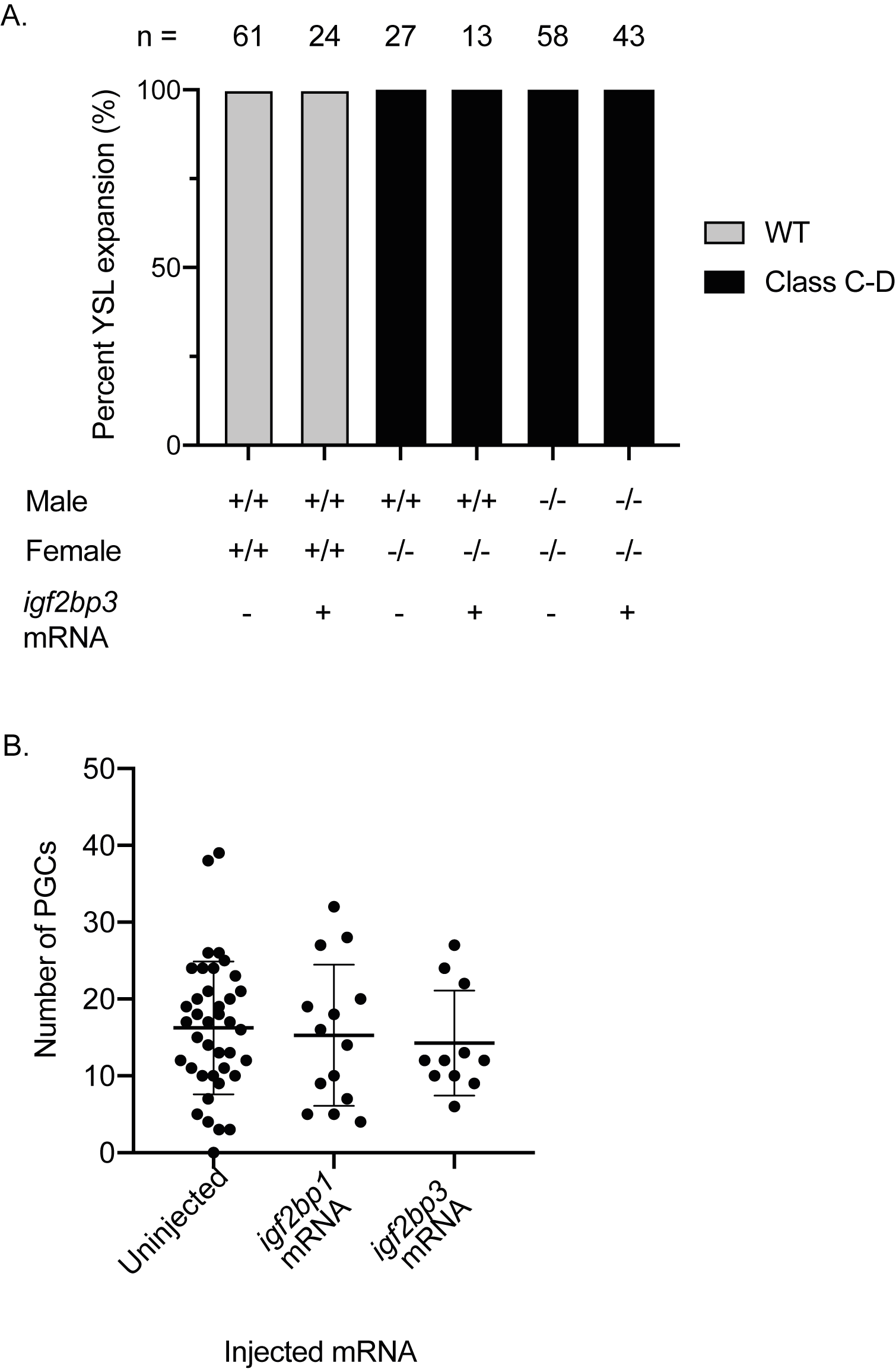

Supplement: S4 Fig — A. Injection of igf2bp3 mRNA into igf2bp3Δ7 embryos does not rescue YSL expansion or lethality. WT, Migf2bp3Δ7 and MZigf2bp3Δ7 embryos were injected with 200 pg of igf2bp3 mRNA at the 1-cell stage and the YSL phenotype scored at 3 hpf. B. Injection of igf2bp1 or igf2bp3 mRNA into igf2bp3la659Tg does not rescue loss of PGCs. MZigf2bp3la659Tg embryos were injected with 100 pg of igf2bp1 or igf2bp3 mRNA at the 1-cell stage and the number of PGCs quantified at 24 hpf. (TIF) [file pgen.1009667.s004.tif]

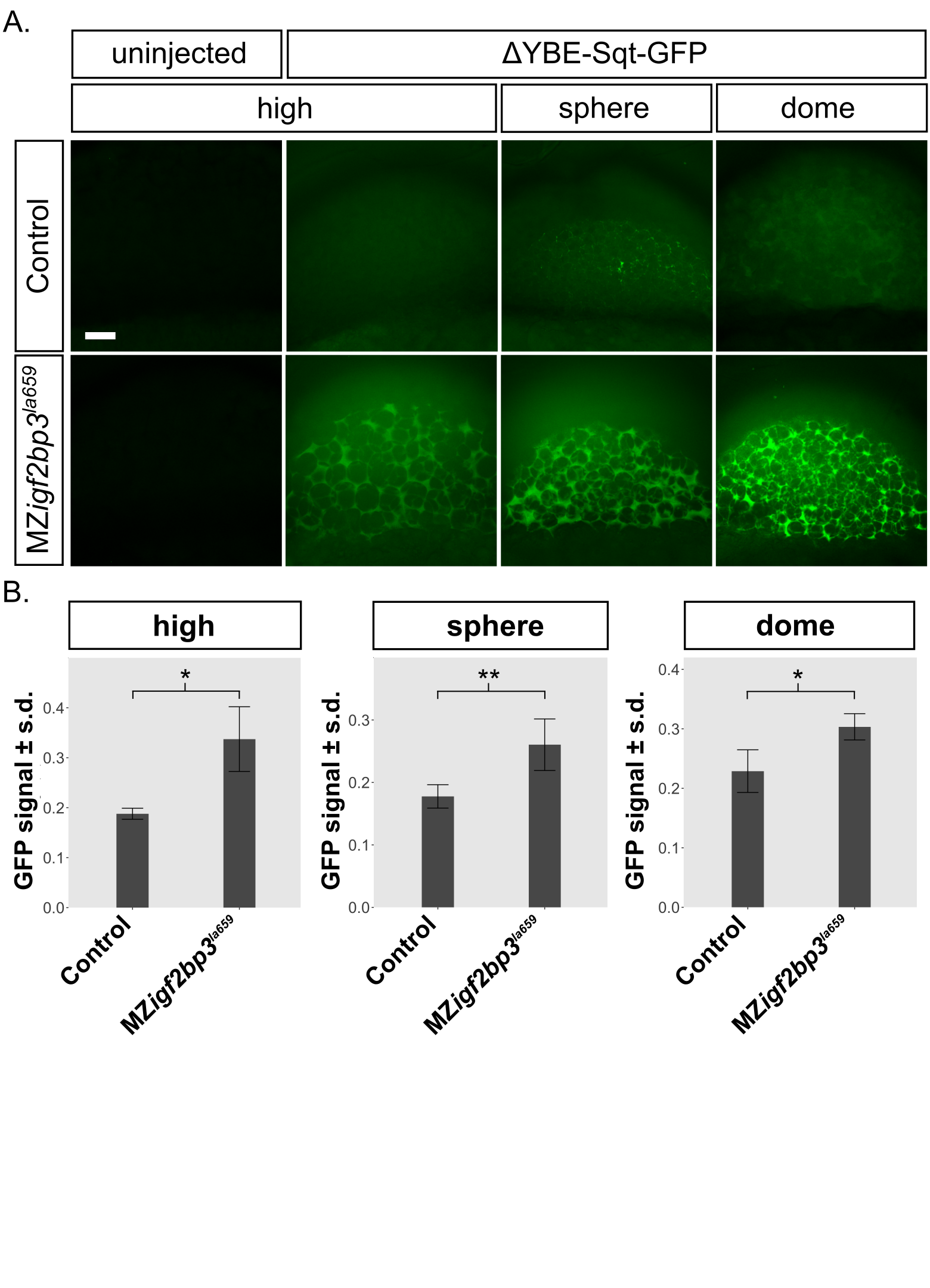

Supplement: S5 Fig — A. ΔYBE Sqt-GFP reporter expression (green signals in extracellular space) is premature and elevated in MZigf2bp3la659Tg embryos compared to control embryos at the high, sphere and dome stages. B. Bar graphs show mean GFP signal intensity in the blastoderm of imaged embryos. GFP levels in the blastoderm were normalised to levels of co-injected rhodamine dextran control. Representative examples from three independent experiments are shown. Number of embryos analysed: high (N = 3 WT and 4 MZigf2bp3la569Tg), sphere (N = 6 control and 7 MZigf2bp3la569Tg), dome (N = 4 WT and 5 MZigf2bp3la569Tg). Asterisks indicate level of significance from two-tailed t tests; p *<0.05, **<0.01, ***<0.001. Scale bar, 50 μm. (TIF) [file pgen.1009667.s005.tif]

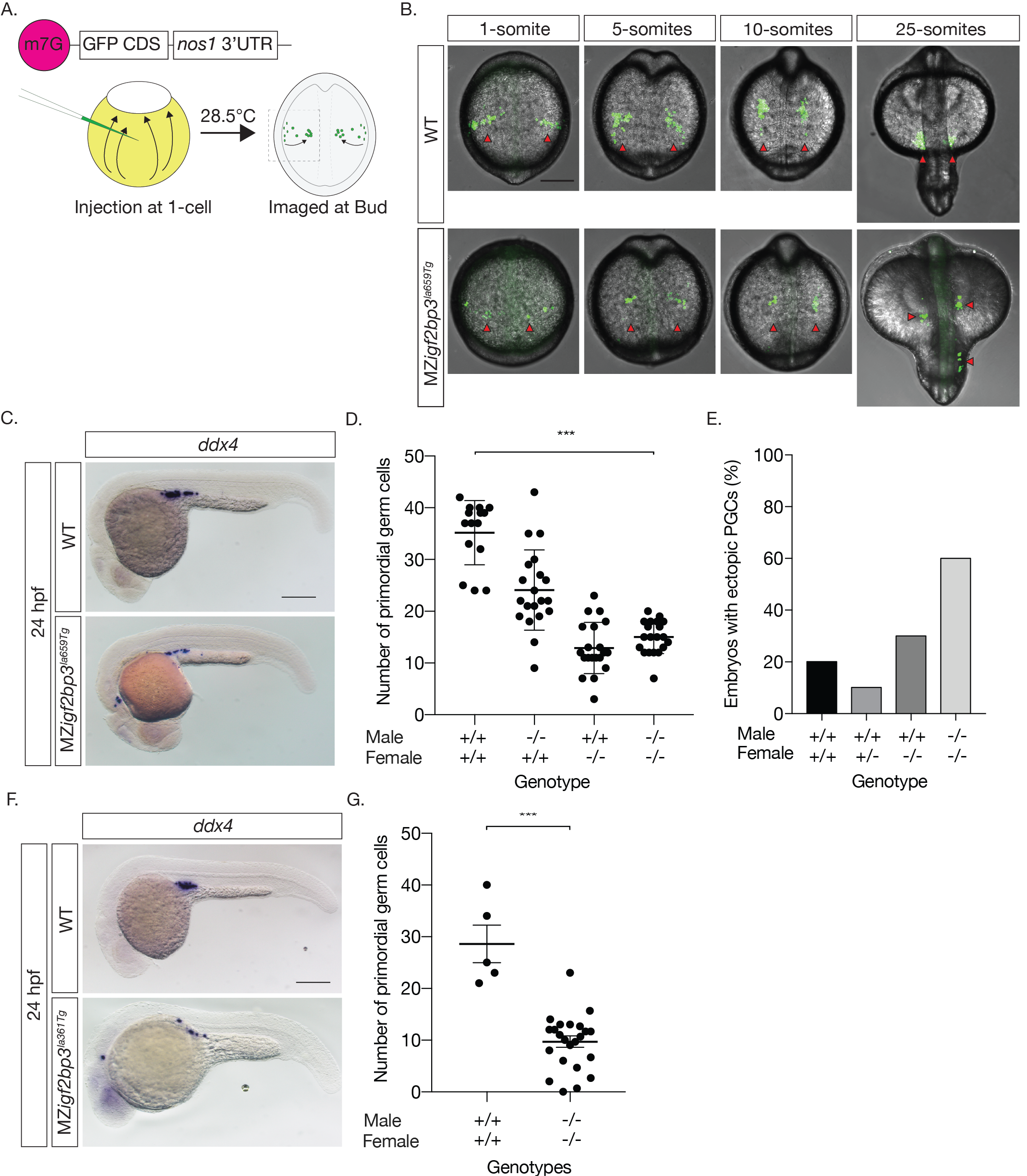

Supplement: S6 Fig — A,B. Live imaging of PGCs in embryos injected with GFP-nos3’UTR reporter mRNA shows reduced PGC numbers in igf2bp3la659Tg mutants compared to WT embryos at 1-somite, 5-somite, 10-somites and 25-somites. C-E. PGC numbers are reduced and germ cells are ectopically located in MZigf2bp3la659Tg mutants (C, E), and reduced in both Migf2bp3la659Tg and MZigf2bp3la659Tg embryos (D). F. The germline is also mis-regulated in a second transgenic insertion mutant line, igf2bp3la361Tg with significantly reduced PGCs in MZigf2bp3la361Tg embryos (F, G). N = 15 WT, 20 Pigf2bp3la659Tg 20 Migf2bp3la659Tg and 25 MZigf2bp3la659Tg embryos in C-E, and 5 WT and 23 MZigf2bp3la361Tg mutants in F,G. Scale bars in C and F, 200 μm; * p<0.05, **<0.01, ***<0.001. (TIF) [file pgen.1009667.s006.tif]

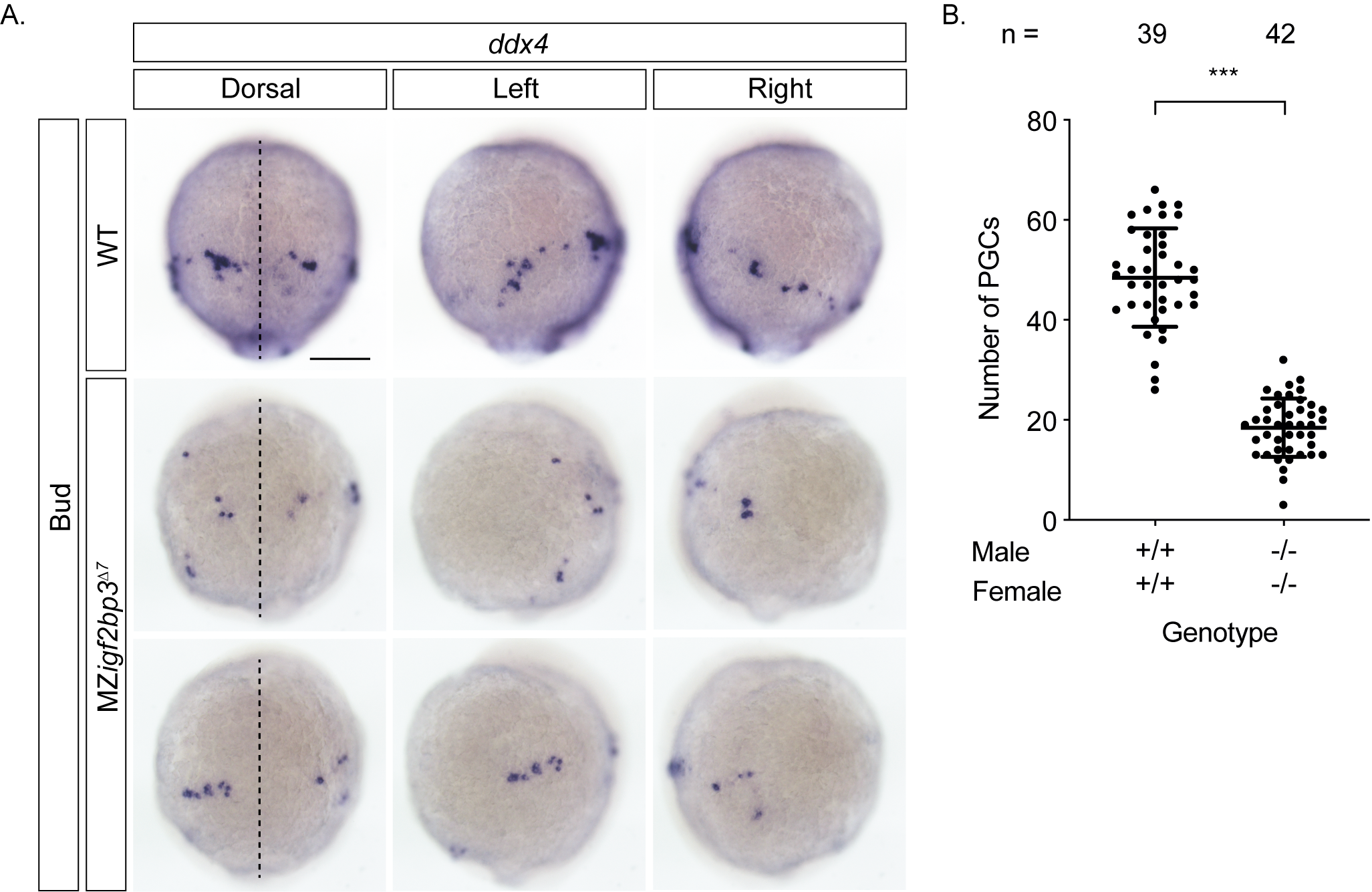

Supplement: S7 Fig — A,B. WISH to detect the germline marker ddx4 (A) shows that PGCs are reduced (quantitation in B), and ectopically located relative to the midline (dashed line) in Migf2bp3Δ7 embryos by Bud stage. N = 39 WT and 42 MZigf2bp3Δ7 mutants; Scale bar in A, 200 μm; p* < 0.05, ** < 0.01, *** < 0.001. (TIF) [file pgen.1009667.s007.tif]

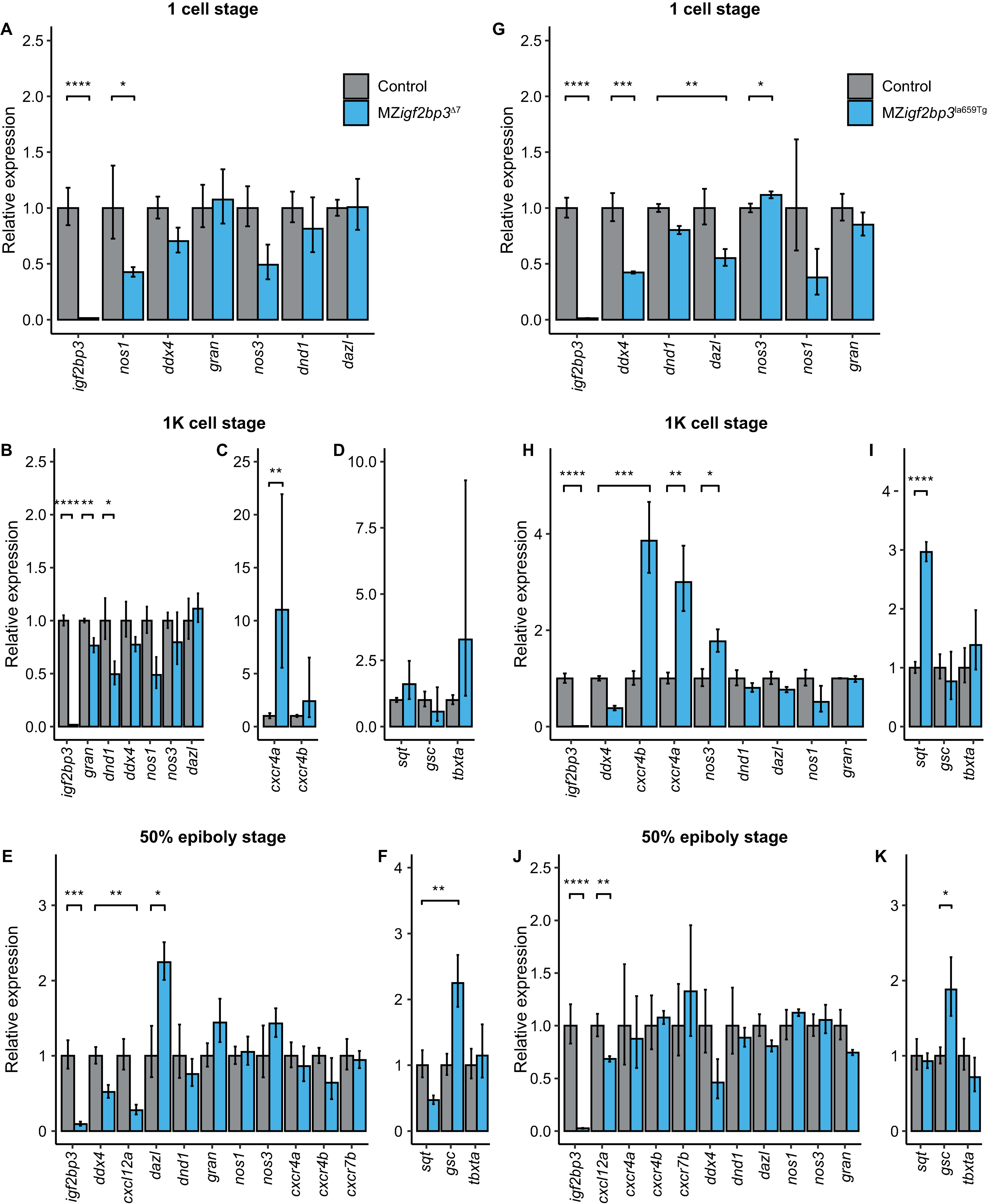

Supplement: S8 Fig — A-F. qRT-PCR of Migf2bp3Δ7, MZigf2bp3Δ7 or MZigf2bp3Δ7 in a Tg(buc:buc-egfp); bucp106+/- background and MZigf2bp3la659Tg (G-K) mutant embryos compared to wildtype controls, shows variable reduction in nos1, ddx4, dnd1 and dazl expression in one-cell stage mutant embryos. Reduction in ddx4, nos1, dnd1 and gran is observed in MZigf2bp3Δ7 and MZigf2bp3la659Tg embryos at the 1K stage. C, H. At the 1K stage, expression of the chemokine receptors, cxcr4a and cxcr4b, is elevated in igf2bp3 mutant embryos, whereas at 50% epiboly (E, J) ddx4 and cxcl12a levels are reduced in the mutants. At 1K-cell stage, there is an increase in sqt and a variable increase in tbxta in both mutants (D, I). At 50% epiboly (F, K), sqt is significantly reduced or reverts to normal levels in MZigf2bp3la659Tg and MZigf2bp3Δ7 embryos, respectively; gsc is significantly increased in both mutants. Data from 3 biological replicates of 25–50 embryos each; p *<0.09, **<0.05, ***<0.01, ****<0.001. (TIF) [file pgen.1009667.s008.tif]
