# Supplementary material for: The RNA-binding protein Igf2bp3 is critical for embryonic and germline development in zebrafish
Source: PLoS Genet. 2021 Jul 2;17(7):e1009667. doi: 10.1371/journal.pgen.1009667 (PMC8282044; doi:10.1371/journal.pgen.1009667)
Supplement: S1 Methods — Primers for qRT-PCR, genotyping, and sequence of aptamers for tobramycin affinity pull-downs. (DOCX) [file pgen.1009667.s017.docx]

**S1 Methods**

Included is the list of primers for qRT-PCR, genotyping, and list of sequences for the aptamer for tobramycin pulldowns.

**List of qRT-PCR primers**

| Gene | Primer name | Sequence (5’ – 3’) | Amplicon length (bp) |
| --- | --- | --- | --- |
| *18S* | 18S qPCR F | TCGCTAGTTGGCATCGTTTATG | 62 |
| *18S* | 18S qPCR R | CGGAGGTTCGAAGACGATCA |  |
| *dnd1* | Dead end  forward | AGATGGACTTCCTTCTCCAAGTC | 230 |
| *dnd1* | Dead end reverse | ATCAGCTCATTTCTTGACATTATGG |  |
| *nos1* | Nanos1 forward | GCTCTTCTGTTCAGCTCGAG | 414 |
| *nos1* | Nanos1 reverse | GGCACCTCATGCAGAAGAATAGC |  |
| *ddx4* | Vasa  forward | CAACAGCAAGGAAAATATAGTCCA | 192 |
| *ddx4* | Vasa reverse | ATCTAGTTCTGGATGAAGCAGACAG |  |
| *igf2bp3* | Igf2bp3 RT-PCR Ex13 F | ATTGCGCCTGCTGATGGAAT | 207 |
| *igf2bp3* | Igf2bp3 RT-PCR Ex4 R | CGTTTTGCCTCCTTTACCAATG |  |
| cxcr7b | cxcr7b F | GAGCACCAAAACACATCGTCA | 109 |
| cxcr7b | cxcr7b R | TCAGTTCACCCAGTGCATCC |  |
| *mxtx2* | Mxtx2 Fw | TCTGATCTGCAAGCAACACC | 232 |
| *mxtx2* | Mxtx2 Rev | TGTCCCAAAATGCAGAATCA |  |
| *camsap3* isoform X4 | camsap3_X4_F | CACTCTTACAGCTCCTGGGC | 332 |
| *camsap3* isoform X4 | camsap3_X4_R | CCTGTACCGGATCGAGGGTT |  |
| *dazl* | ZdazlF | TACCCGTGTGCCTGATATGTGG | 374 |
| *dazl* | ZdazlR | TGACACTGACCGAGAACTTCGC |  |
| *nanos3* | nos fw | TGGATCTATGGAGACTAGAAACCAG | 246 |
| *nanos3* | nos rev | CAGAAGCTGCAGAACTTTCTCTCT |  |
| *granulito* | Gra fw | GAAAAACCCGACAACATTATTACAG | 241 |
| *granulito* | Gra rev | TGTTGTTGTAACTGGTGGAGAAGTA |  |
| *cxcr4a* | cxcr4a f | GGCTTATTACGGACACATCGTC | 340 |
| *cxcr4a* | cxcr4a r | CATGAACCCTCCAAAGTACCAGTC |  |
| *cxcr4b* | cxcr4b f | GGACTTGTGGTGCTTGTGATG | 403 |
| *cxcr4b* | cxcr4b r | GGTAAGTAAGCTCGCAGATGG |  |
| *cxcl12a* | cxcl12a_Fw | ATGACCTGATTCTGCTGAGCGTGA | 146 |
| *cxcl12a* | cxcl12a_Rev | TGGCTTCACTTGAAGGGTCGATTG |  |
| *sqt/ndr1* | sqt-Ex2-F | GAACCACAGAACTGATGATA | 231 |
| *sqt/ndr1* | sqt-Ex2-R | GCATGGTTTGTTGGAGTGAA |  |
| *gsc* | gsc Ex1 Fw | TGGAAGGATAGGCTACAACAACTAC | 320 |
| *gsc* | gsc Ex2 Rev | GGTATTTCGTTTCTTGAAAAAGGTT |  |
| *ntl/tbxta* | NtlEx3 Fw | TATTGCAGTCACAGCATATCAGAAT | 270 |
| *ntl/tbxta* | Ntl Ex6 Rev | AAGCTGGAGTATCTCTCACAGTACG |  |

**Genotyping primers**

| Allele | Primer name | Sequence (5’ – 3’) |
| --- | --- | --- |
| *igf2bp3^∆7^* | Igf2bp3 geno/seq PCR F | ATCCCATGGATGAATAAGCTGTACATC  GGG |
| *igf2bp3^∆7^* | Igf2bp3 geno/seq PCR R | GATCATATGGCACACAATCCCTGCTTT  GCAT |
| *igf2bp3^la010361Tg^* | 5071 | GATGAGGGCCATTGACACGC |
| *igf2bp3^la010361Tg^* | 5070 | GCAACGCTGCAACTTTCATAGACC |
| *igf2bp3^la020659Tg^* | 5069 | GTCTCGCTGCTCGTGGCTAGG |
| *igf2bp3^la020659Tg^* | 5068 | CTTAGGGACAGAGTGCTCCACTTC |
| Transgenic insertion allele common primer | 3’ LTR F22 | AAAGACCCCACCTGTAGGTTTG |

**Aptamers for Tobramycin pull down**

**Aptamer sequence**

ATGCTAGCGGGAGAAGACGACCGACCAGAATCATGCAAGTGCGTAAGATAGTCGCGGGCCGGGAAAAAAAAAAGGCTTAGTATAGCGAGGTTTAGCTACACTCGTGCTGAGCCAAAAAAAAAAGACCGACCAGAATCATGCAAGTGCGTAAGATAGTCGCGGGCCGGGTATGTGCGTCTG

***ndr1/sqt* 5'UTR: aptamer: *ndr1/sqt* 3'UTR**

ACGAGCTTTATTTCAATAACTGCGTGTGGATTATTACCTTGATTTGACATGTTTTCCTGCGGGCTCCTGAGCGTAGTTTTGGCCCTTATGCTAGCGGGAGAAGACGACCGACCAGAATCATGCAAGTGCGTAAGATAGTCGCGGGCCGGGAAAAAAAAAAGGCTTAGTATAGCGAGGTTTAGCTACACTCGTGCTGAGCCAAAAAAAAAAGACCGACCAGAATCATGCAAGTGCGTAAGATAGTCGCGGGCCGGGTATGTGCGTCTGGATCCTATTGGCAAGATGGTCATGAGACACCATGAAGGCATGGTTGTTGCAGAATGCGGCTGCCACTGATTCTTCAAACCCCAAAGGAACTCAACTCTAGCACTTTGGATATGCTCCTTGACCCCAAAAATATGTATTTAAGAAAAACTGCTGTCAATTATTCCCACTTGAAATTATTATGGTTTCCTGCACTGAGGCACCTGGATAACTTGATGCTATTATTGAAAGCTTTGCGTGTTTGCCTTATCTGTAAATAGTAGAGTATGTAAATTACCAAATGTAATAAAATGTTTTCATAATGTTTAAAAAAAAAAAAAAAAAA

**Aptamer: *ndr1/sqt* 3'UTR**

TATGCTAGCGGGAGAAGACGACCGACCAGAATCATGCAAGTGCGTAAGATAGTCGCGGGCCGGGAAAAAAAAAAGGCTTAGTATAGCGAGGTTTAGCTACACTCGTGCTGAGCCAAAAAAAAAAGACCGACCAGAATCATGCAAGTGCGTAAGATAGTCGCGGGCCGGGTATGTGCGTCTGGATCCTATTGGCAAGATGGTCATGAGACACCATGAAGGCATGGTTGTTGCAGAATGCGGCTGCCACTGATTCTTCAAACCCCAAAGGAACTCAACTCTAGCACTTTGGATATGCTCCTTGACCCCAAAAATATGTATTTAAGAAAAACTGCTGTCAATTATTCCCACTTGAAATTATTATGGTTTCCTGCACTGAGGCACCTGGATAACTTGATGCTATTATTGAAAGCTTTGCGTGTTTGCCTTATCTGTAAATAGTAGAGTATGTAAATTACCAAATGTAATAAAATGTTTTCATAATGTTTAAAAAAAAAAAAAAAAAA
